# Nonthermal Pretreatment Technologies to Improve Drying Efficiency and Quality in Fresh-Cut Fruits and Vegetables: A Comprehensive Review

**DOI:** 10.3390/foods15030568

**Published:** 2026-02-05

**Authors:** Nemanja Miletić, Alexander Lukyanov, Marko Petković

**Affiliations:** 1 Faculty of Agronomy in Čačak, University of Kragujevac, Cara Dušana 34, 32102 Čačak, Serbia; n.m.miletic@kg.ac.rs; 2 Department of Automation of Production Processes, Don State Technical University, Gagarin sq. 1, 344002 Rostov-on-Don, Russia; alexlukjanov1998@gmail.com

**Keywords:** fresh-cut, pulsed electric field, ultrasound, cold plasma, osmotic dehydration, high-pressure processing, freeze-thaw, combined pretreatments

## Abstract

The preservation of fresh-cut fruits and vegetables through dehydration is undergoing a paradigm shift to overcome quality degradation and high energy intensity associated with conventional thermal drying. This review synthesizes advancements in innovative pretreatments, focusing on their mechanisms, synergistic effects, and industrial readiness. Non-thermal pretreatment (NTP) methods—including Pulsed Electric Fields (PEF), Ultrasound (US), Cold Plasma (CP), and High-Pressure Processing (HPP)—are evaluated alongside optimized Osmotic Dehydration (OD) and Freeze-Thaw (FT) cycles. Analysis reveals these technologies enhance drying kinetics, reducing processing time by 20–55%, while improving bioactive retention by 30–95%. A critical discussion of Technology Readiness Levels (TRL) distinguishes commercially mature solutions like OD (TRL 9) and HPP (TRL 8–9) from emerging pilot-scale concepts like US and PEF (TRL 6–7). Cold Plasma remains at TRL 4–5 due to uniformity challenges. Furthermore, the higher capital expenditure of innovative systems is mitigated by operational energy savings (up to 50%) and “clean label” premiums. This paper provides a strategic framework to optimize pretreatment selection based on tissue matrices and economic viability.

## 1. Introduction

Fresh commodities, particularly fruits, vegetables, meats, and aquatic products, possess high perishability due to their elevated moisture content and nutrient-dense matrices, which facilitate rapid microbial proliferation and deleterious enzymatic or oxidative reactions. Dehydration serves as a fundamental preservation strategy by systematically reducing moisture content and water activity (a_w_), thereby inhibiting spoilage microorganisms and oxidative degradation while theoretically retaining essential nutrients to extend product shelf life [1]. While drying remains one of the oldest and most indispensable food preservation techniques globally, conventional thermal methods—such as hot air drying—often lead to prolonged processing durations, excessive energy footprints, and the significant degradation of thermosensitive nutritional and sensory profiles [2,3].

To mitigate these limitations, pretreatment is recognized as a vital preparatory step for drying operations, widely employed to inactivate browning enzymes, modify tissue microstructure, and facilitate accelerated moisture removal [4]. The strategic application of these methods can significantly curtail drying time and energy consumption while concurrently enhancing the chemical and physical integrity of the final dried product [5,6].

These strategies are broadly categorized by their thermodynamic and mechanical impacts:

- Conventional methods: Chemical dipping and thermal blanching traditionally utilize osmotic gradients or heat to alter cellular permeability and inactivate enzymes.
- Non-thermal technologies: Advanced systems utilize physical forces to achieve cellular disruption without heat. Pulsed Electric Fields (PEF) utilize electroporation to create nano- to micrometer pores in cell membranes. Ultrasound (US) employs acoustic cavitation and microstreaming to disrupt cell walls and reduce boundary layer resistance. Cold Plasma (CP) utilizes ROS and RNS species for surface etching and enzyme oxidation. HPP induces volumetric structural changes and protein unfolding to alter tissue density.

Drying is an energy-intensive process; however, it can be significantly optimized through pretreatments that enhance mass transfer and minimize the total energy load required for water evaporation [7,8]. Various drying platforms—including convective hot air, vacuum, freeze-drying, and microwave-assisted systems—each present unique resistance to moisture diffusion. Recent literature has focused on the mechanical benefits of emerging pretreatment approaches, specifically Ultrasound (US), High-Pressure Processing (HPP), and Pulsed Electric Fields (PEF), emphasizing their ability to improve mass transfer kinetics and safeguard quality attributes [9,10]. Despite these advancements, there is a need for a critical synthesis that evaluates how these technologies integrate with modern drying processes across diverse fresh-cut matrices.

By examining fundamental mechanisms, product-specific optimizations, and the Technology Readiness Levels (TRL) of each approach, this article establishes a framework for sustainable, quality-oriented food processing. The maturity and industrial readiness of various pretreatment technologies for the fresh-cut drying industry can be categorized by their TRL, ranging from established commercial standards to emerging experimental methods [1,2,3,4,5,6,7,8,9,10]. OD stands as a fully mature technology (TRL 9), seeing extensive industrial implementation, especially within the tropical fruit sector. Similarly, High-Pressure Processing (HPP) has achieved commercial stability (TRL 8–9); however, while it is a benchmark for microbial safety, its specific utilization as a drying pretreatment is currently constrained by significant capital expenditure requirements. Both PEF and US, demonstration and pilot stages (TRL 6–7), have advanced to the demonstration phase. While they garner increasing industrial interest for their ability to enhance drying kinetics, their widespread adoption is currently hindered by technical hurdles related to equipment scale-up and maintaining field uniformity across large batches. FT pretreatments are currently at a pilot level (specialized and niche applications, TRL 5–6). Although they are highly effective at modifying the texture of specific products, such as carrot slices, their application remains limited to niche markets due to the specific structural requirements of the produce. CP, experimental and laboratory research (TRL 4–5), remains in the early experimental stages regarding its role in the drying industry. Its transition to higher TRLs is currently stalled by fundamental challenges in designing equipment capable of ensuring uniform treatment across sliced produce surfaces.

The objective of this comprehensive review is to provide an up-to-date evaluation of innovative non-thermal pretreatment technologies applied to fresh-cut fruits and vegetables before dehydration.

## 2. Nonthermal Pretreatment Technologies

### 2.1. Pulsed Electric Field Pretreatment

#### 2.1.1. Mechanism and Principles

Pulsed electric field (PEF) is a non-thermal food processing technology that induces electroporation of the cell membrane, thus improving mass transfer through the cell membrane [11,12]. The application of short, high-voltage pulses (typically 0.1–80 kV cm^−1^) to food materials placed between two electrodes causes the formation of pores in cell membranes, a phenomenon known as electroporation or electro-permeabilization [13,14]. This structural modification enhances the permeability of plant tissues, facilitating moisture removal during subsequent drying processes [15]. The PEF treatment parameters that influence the degree of electroporation include electric field strength, pulse width, pulse number, pulse frequency, and treatment time. The energy input during PEF treatment is typically expressed in kJ kg^−1^ and ranges from 1 to 100 kJ kg^−1^ for most food applications [16]. The extent of cell membrane permeabilization can be quantified by measuring the electrical conductivity of the tissue or by using the disintegration index (Z_p_), which ranges from 0 (intact tissue) to 1 (completely disintegrated tissue) [17].

#### 2.1.2. Effects on Drying Characteristics

Numerous studies have demonstrated the effectiveness of PEF pretreatment in reducing drying time and enhancing drying kinetics. Kim et al. [18] reported that PEF pretreatment reduced the drying time of carrot by up to 28% at 50 °C. The study concluded that PEF resulted in an effective pretreatment for carrots before undergoing convective drying because it reduced drying time and, particularly at mild temperatures (50 °C), did not affect the texture properties of both vegetables. PEF pretreatment at 2.5 kV cm^−1^ showed the shortest drying time, taking 180 min, whereas the control required 330 min for the same moisture ratio, indicating a 45% reduction in drying time. In PEF pretreatment, the drying time of apple slices was reduced by 37–57% in comparison to untreated apples slices [19,20].

The enhanced drying kinetics observed with PEF pretreatment can be attributed to the increased tissue permeability, which facilitates moisture migration from the interior to the surface of the product. The effective moisture diffusivity (D_eff_) values have been reported to increase by 1.5 to 3 times with PEF pretreatment compared to untreated samples [13].

#### 2.1.3. Quality Attributes

A literature search revealed that PEF-assisted drying is beneficial for maintaining the physicochemical properties of the dried products and preserving their color and constituent chemical compounds. PEF-assisted drying promotes rehydration and improves the kinetics of drying. After the drying and rehydration process, the change in color of PEF-pretreated samples was less significant compared to untreated ones [18,21].

One of the most significant advantages of PEF pretreatment is its ability to enhance the retention of bioactive compounds. Liu et al. [22] reported that the difference in carotenoid content of rehydrated carrots between the control and 2.5 kV cm^−1^ PEF-treated samples was more than 2.5 times. The application of 3–5 kV cm^−1^ pretreatment of PEF increased the total carotenoid content due to the disruption of cellular structure and ROS generation by the stress-response mechanism of the plant [23]. Furthermore, after treatment of PEF (five pulses, 3.5 kV cm^−1^, 0.61 kJ kg^−1^), carotenoid content increased to more than 80% compared to untreated carrots [24].

The improved retention of bioactive compounds with PEF pretreatment has also been reported for polyphenols, anthocyanins, and vitamin C in various fruits and vegetables [25]. The mechanism behind this enhanced retention is attributed to the reduced thermal exposure time during drying (due to faster drying rates) and the potential protective effect of cell membrane disruption, which may reduce enzymatic degradation [26,27].

Generally speaking, PEF applies short bursts of high-voltage electricity to fruit and vegetable tissues, inducing a phenomenon known as electroporation.

- Physical: The primary physical impact is the increased permeability of cell membranes. This facilitates faster moisture loss during subsequent drying, often resulting in a more porous structure and reduced shrinkage compared to untreated samples.
- Chemical: PEF is a non-thermal process, which helps in the high retention of thermolabile compounds like Vitamin C and anthocyanins. However, excessive field strength can lead to some electrochemical reactions that may slightly alter antioxidant profiles.
- Sensory: Consumers generally perceive PEF-treated products as having a fresher taste and better color retention because the low-heat approach prevents the Maillard reaction. Texture is often described as less “rubbery” due to uniform moisture removal.

#### 2.1.4. Energy Efficiency

From the viewpoint of economic sustainability, PEF technology enables food processing to be conducted at a lower cost in terms of energy [28]. Continuous application of PEF has been indicated to be more energy-efficient than most thermal treatments by a large body of literature. Moreover, PEF pretreatment is considered particularly promising for the industrial sector because of less energy consumption compared to conventional thermal pretreatments [29,30].

The total energy consumption for drying PEF-pretreated samples is lower than for untreated samples, despite the additional energy required for the PEF treatment itself [31]. This is because the reduction in drying time more than compensates for the energy used during PEF application. Wiktor et al. [32,33] reported that the total SEC for convective drying of PEF-pretreated apples was reduced by approximately 20% compared to untreated samples.

#### 2.1.5. Advantages and Disadvantages of PEF Pretreatment

Table 1 shows the advantages and disadvantages of ultrasonic pretreatment of fruits and vegetables before drying.

**Figure 1:**
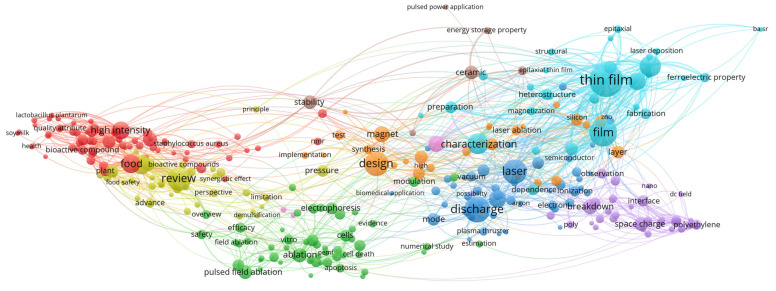
Co-occurrence analysis of terms (PEF) related to “NTP nonthermal pretreatment” was performed based on titles, abstracts, and keywords. The size of each circle indicates the frequency of term occurrence, while different colors represent distinct clusters of closely related keywords, enabling their classification. The term map was generated using VOSviewer software, with data obtained from the Scopus database.

**Figure 2:**
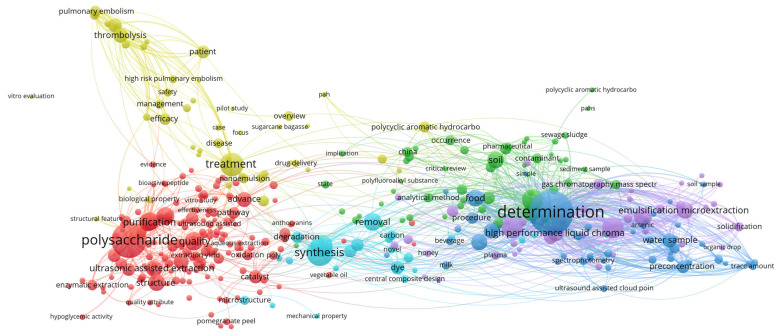
Co-occurrence analysis of terms (US) related to “NTP nonthermal pretreatment” was performed based on titles, abstracts, and keywords. The size of each circle indicates the frequency of term occurrence, while different colors represent distinct clusters of closely related keywords, enabling their classification. The term map was generated using VOSviewer software, with data obtained from the Scopus database.

**Figure 3:**
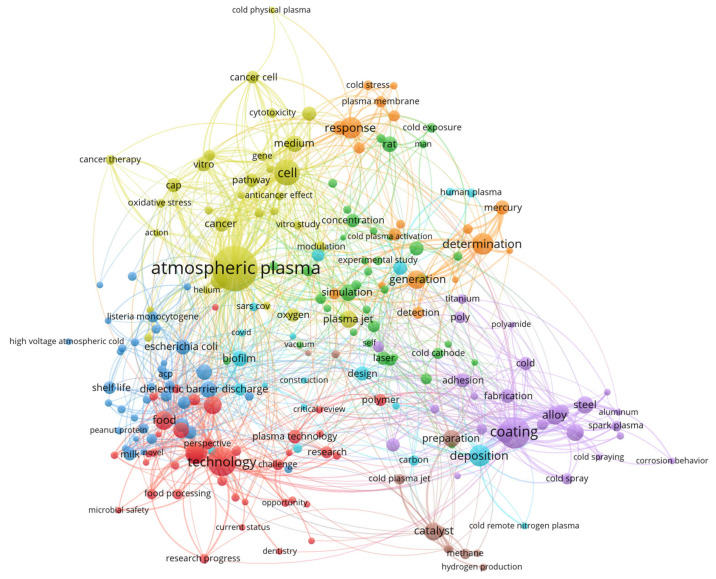
Co-occurrence analysis of terms (CP) related to “NTP nonthermal pretreatment” was performed based on titles, abstracts, and keywords. The size of each circle indicates the frequency of term occurrence, while different colors represent distinct clusters of closely related keywords, enabling their classification. The term map was generated using VOSviewer software, with data obtained from the Scopus database.

**Figure 4:**
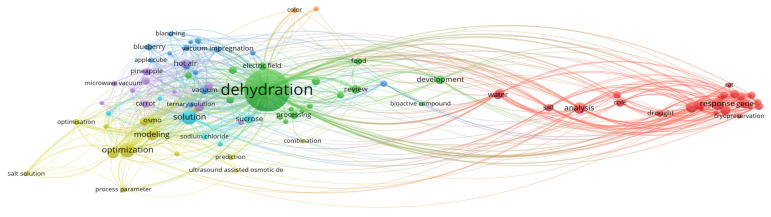
Co-occurrence analysis of terms (OD) related to “NTP nonthermal pretreatment” was performed based on titles, abstracts, and keywords. The size of each circle indicates the frequency of term occurrence, while different colors represent distinct clusters of closely related keywords, enabling their classification. The term map was generated using VOSviewer software, with data obtained from the Scopus database.

**Figure 5:**
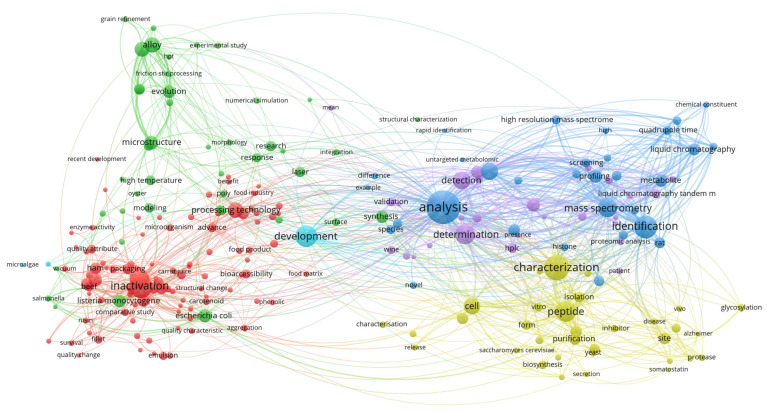
Co-occurrence analysis of terms (HPP) related to “NTP nonthermal pretreatment” was performed based on titles, abstracts, and keywords. The size of each circle indicates the frequency of term occurrence, while different colors represent distinct clusters of closely related keywords, enabling their classification. The term map was generated using VOSviewer software, with data obtained from the Scopus database.

**Figure 6:**
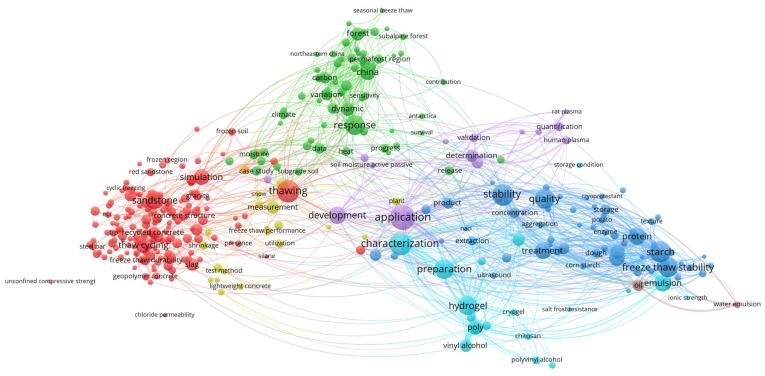
Co-occurrence analysis of terms (FT) related to “NTP nonthermal pretreatment” was performed based on titles, abstracts, and keywords. The size of each circle indicates the frequency of term occurrence, while different colors represent distinct clusters of closely related keywords, enabling their classification. The term map was generated using VOSviewer software, with data obtained from the Scopus database.

**Figure 7:**
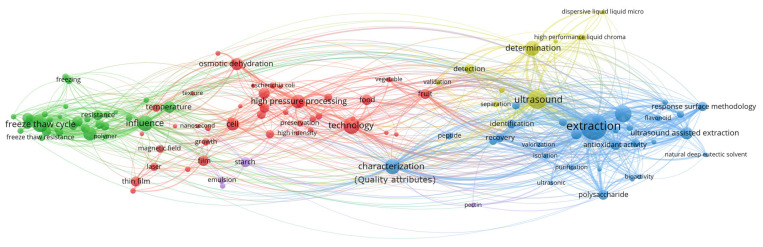
The unique co-occurrence analysis of different pretreatment terms related to “NTP nonthermal pretreatment” was performed based on titles, abstracts, and keywords (Figure 1, Figure 2, Figure 3, Figure 4, Figure 5 and Figure 6). The size of each circle indicates the frequency of term occurrence, while different colors represent distinct clusters of closely related keywords, enabling their classification. The term map was generated using VOSviewer software, with data obtained from the Scopus database.

To construct this review, data were aggregated from the published literature and major electronic databases, including Web of Science, Scopus, and Google Scholar. The search strategy targeted using the term “nonthermal pretreatment” in conjunction with specific technologies such as PEF, US, CP, OD, HPP and FT. Selection was limited to food technology, focused on drying methods that included at least one NTP. Previously published review papers were excluded from the analysis. To map research trends within the field of NTP, titles, abstracts, and keywords were processed using VOSviewer (version 1.6.20). As illustrated in the Figure 1, Figure 2, Figure 3, Figure 4, Figure 5, Figure 6 and Figure 7, the bibliometric analysis of these abstracts identified different primary clusters related to the study of nonthermal pretreatments.

The bibliometric co-occurrence diagram reveals a highly concentrated research landscape within the food technology sector, primarily localized in the red and yellow clusters on the left of the map (Figure 1). In this domain, the diameter of each circle serves as a direct indicator of keyword frequency, where dominant nodes such as “food”, “high intensity” and “review” appear as the largest circles, identifying them as the foundational anchors of the field. The correspondence between circle size and color signifies a thematic hierarchy: the red cluster acts as the primary hub for biological and chemical interactions, encompassing keywords like “bioactive compound”, “quality attribute” and “plant” while the yellow cluster shifts toward evaluative and regulatory frameworks, aggregating terms like “safety”, “efficacy” and “advance.” This color-coded separation illustrates how research is partitioned between the fundamental application of pulses to food matrices and the subsequent validation of those processes for industrial safety.

The aggregation of terms within these clusters highlights the multidisciplinary nature of nonthermal pretreatment research. Within the red cluster, the tight grouping of “high intensity” with “bioactive compounds” and “plant” illustrates the specific use of PEF as a nonthermal tool to enhance the extraction of phytochemicals or to stabilize sensitive nutrients without the use of heat.

This is further supported by the co-occurrence of specific microbial targets such as “*Lactobacillus plantarum*” and “*Staphylococcus aureus*” around the central “food” node, which underscores the technology’s dual role in nonthermal pasteurization and shelf-life extension. The spatial proximity and dense web of lines connecting “food safety” to “efficacy” and “synergistic effect” suggest that modern food technology research is increasingly focused on combining PEF with other nonthermal hurdles to maximize microbial inactivation while maintaining the sensory and “health” attributes of the product.

Interrelationships based on correlations between keywords extend beyond simple classification, showing that “stability” and “characterization” serve as vital conceptual bridges between the biological food clusters and the broader engineering aspects of the diagram. The presence of peripheral nodes like “soymilk” and “health” indicates specialized application areas that are highly correlated with the core “food” node, suggesting that PEF is being tailored for specific functional beverage categories.

Detailed graphical diagrams (Figures S1–S6) illustrating the underlying physical mechanisms have been added as Supplementary Files.

### 2.2. Ultrasound Pretreatment

#### 2.2.1. Mechanism and Principles

Due to its non-thermal character, ultrasound can be a potential substitute for heat-based pretreatments such as blanching, microwave and infrared [41]. Ultrasound with high intensity (>1 W cm^−2^) induces a phenomenon known as cavitation, which is useful to remove moisture that is firmly bound to cells [42]. Cavitation can be achieved through the use of ultrasound frequencies typically in the range of 20–100 kHz. Ultrasound devices are capable of producing acoustic cavitation, which is the formation, growth, and collapse of air bubbles within a system [43,44]. A significant challenge in the industrialization of US is the non-uniform acoustic field distribution within large-scale sono-reactors. Due to acoustic attenuation and the formation of standing waves, ‘shadow zones’ can emerge where cavitation intensity is insufficient for cellular disruption. Recent advancements suggest that utilizing centrally symmetric multi-source configurations and real-time finite element method simulations are essential to ensure treatment consistency and avoid localized enzymatic browning in fresh-cut matrices [43,44].

The travel of ultrasonic waves through a medium lead to a series of compression and rarefaction waves similar to a sponge effect. The sponge effect occurs when ultrasonic waves travel through fruit or vegetable tissue, causing rapid alternating compression and expansion of the tissue. This phenomenon generates microscopic channels that facilitate removal of moisture, as water molecules use these channels to move towards the surface of fruits and vegetables [45,46].

US pretreatment can be applied using two main configurations: ultrasonic bath (indirect contact) and ultrasonic probe (direct contact). The ultrasonic bath is more suitable for large-scale applications, while the probe system delivers higher energy intensity to the product but is limited in treatment capacity. Key parameters affecting US pretreatment efficiency include frequency, power intensity, treatment time, temperature, and sample characteristics [47,48].

#### 2.2.2. Effects on Drying Performance

The results of various studies have consistently shown that US pretreatment accelerates the drying rate. The advantage is a simultaneous improvement in drying efficiency and product quality, achieved by the optimal US pretreatment range (0.34–0.54 W g^−1^) which significantly shortens the drying time and increases the key functional component, polysaccharide content. This provides crucial data for optimizing the drying process and advancing the industrial application of *Tremella fuciformis* [49].

Wang et al. [50] investigated the effect of low-frequency US pretreatment on carrot slices dried by intermittent infrared drying. Results showed that the vacuole water of this method-pretreated carrot samples decreased, while the cytoplasm and intercellular space water increased, as determined by low-field nuclear magnetic resonance analysis. In addition, low-frequency US pretreatment caused the disruption of cell structures and formation of micro-channels, resulting in a significant (*p* < 0.05) decrease in drying time required. The infrared-dried carrot slices with low-frequency US pretreatment showed higher *β*-carotene content and rehydration ratio compared with control samples [50].

In a study on red chili, Delfiya et al. reported that US pretreatment at different power levels and durations significantly reduced drying time during subsequent vacuum drying. The microstructural analysis revealed that US treatment created pores and channels in the tissue, facilitating moisture migration [51].

#### 2.2.3. Quality Attributes

Overall, applying US to assist the dehydration of fruits and vegetables is a promising way to reduce drying time and obtain nutritious dehydrated products [52]. This non-thermal technology alleviates the oxidation of nutrients, thus offering a favorable perspective to increase the marketability of finished products as public awareness of food quality is surging [53].

The primary advantage is the enhanced drying efficiency and product quality of carrot slices, where US pretreatment significantly reduced drying time by up to 20% and improved the rehydration ratio of the dried product. Specifically, the US pretreatment offered a further benefit by preserving a *β*-carotene content compared to both probe pretreatment and no pretreatment [54]. The selective effect of US parameters on bioactive compound retention highlights the importance of optimization studies [55].

Color preservation is another important quality attribute improved by US pretreatment. The lower thermal exposure during shortened drying times helps maintain the natural color of fruits and vegetables [56]. Texture properties, particularly rehydration capacity, have also been reported to improve with US pretreatment due to better preservation of cellular structure and reduced collapse during drying [57].

US pretreatment utilizes acoustic cavitation—the formation and collapse of microscopic bubbles—to create “micro-channels” in the tissue.

- Physical: Cavitation causes mechanical disruption of cell walls, which significantly enhances mass transfer. This typically leads to a softer texture initially, but a more uniform crunch or firmness after final processing.
- Chemical: US can promote the release of bound phenolic compounds by breaking down cell wall polysaccharides, potentially increasing the measurable antioxidant capacity of the produce.
- Sensory: The cavitation effect can sometimes lead to different flavors if not controlled, but generally, it preserves the natural aroma and bright colors of fruits like strawberries or mangoes by inactivating oxidative enzymes.

#### 2.2.4. Energy Efficiency

The application of US has been widely studied as an environmentally friendly and energy-efficient method to improve the processing of fruits and vegetables. Specifically, it notably enhances the efficiency of subsequent drying processes, which are known to consume a lot of energy in the food industry [58]. Although the ultrasound process itself requires electrical energy, the overall energy balance is often beneficial. The high efficiency of US arises from the non-thermal structural changes it causes. Because the drying time is greatly reduced, the total thermal energy needed is minimized [59].

While the ultrasound process itself requires electrical energy input, the overall energy balance is often favorable. The high efficiency of US stems from the non-thermal nature of the structural changes it induces. Since the subsequent drying time is substantially curtailed, the total thermal energy required is minimized. For instance, the application of US before atmospheric freeze-drying has been shown to reduce the SEC by up to 70% due to faster sublimation and significantly shorter cycle times [60]. Similarly, combining US with convective drying of fruits has been demonstrated to reduce the overall SEC compared to conventional drying methods alone [61].

#### 2.2.5. Advantages and Disadvantages of Ultrasound Pretreatment

Table 2 shows the advantages and disadvantages of US pretreatment of fruits and vegetables before drying.

The research demonstrates that while US pretreatment offers significant advantages in reducing drying time and improving product quality, success depends heavily on optimizing parameters such as power, frequency, sonication time, and temperature control to minimize negative effects like tissue damage and nutrient loss. However, US can be less expensive than treatments like PEF and HPP, which require cost-intensive equipment. In addition, compared to these technologiesUS can be combined with the drying treatment, thus the pretreatment step can be integrated into the drying process itself [69]. This integration possibility, known as US-assisted drying, offers operational advantages and has been extensively studied for various fruits and vegetables [45,70].

The primary research pillars are anchored by the largest nodes, such as “ultrasonic assisted extraction,” “polysaccharide” and “purification”, which signal that the most significant portion of current literature focuses on the recovery and refining of bioactive macromolecules (Figure 2). This correspondence between circle size and its specific color establishes a clear thematic hierarchy; while the color categorizes the research into distinct scientific sub-domains, the node size indicates which concepts serve as the foundational “anchors” for that discipline.

The aggregation of terms into clusters reveals a strategic division of ultrasound applications within food technology. The red cluster is the most prominent, signifying a massive concentration on ultrasonic-assisted extraction and process optimization. Within this group, keywords like “yield”, “recovery” and “kinetics” reflect a drive toward industrial efficiency and the transition from laboratory scale to pilot production. Secondary clusters, often in green, yellow, or blue, aggregate terms related to “structure”, “functional properties” and “antioxidant activity,” highlighting a shift toward understanding the physicochemical modification of food components like proteins and phenolic compounds.

As a leading nonthermal pretreatment, ultrasound research is characterized by the dense co-occurrence of nodes related to acoustic cavitation. The diagram illustrates this through strong links between “ultrasonic assisted extraction” and “bioactive compounds,” reflecting the technology’s ability to disrupt plant cell walls (micro-jets and shockwaves) at near-ambient temperatures. This prevents the thermal degradation of heat-sensitive nutrients, a theme reinforced by the proximity of keywords like “total phenolic content” and “antioxidant capacity”.

Furthermore, the co-occurrence of “inactivation” and “enzymatic browning” suggests that US is increasingly used as a nonthermal pretreatment to stabilize juices and produce “fresh-like” minimally processed products without the need for high-heat pasteurization.

The interrelationships and correlations based on the diagram’s spatial arrangement identify “microstructure” and “characterization” as critical “bridge” keywords. These nodes are often located at the intersection of extraction and modification clusters, suggesting that structural analysis is a universal prerequisite regardless of the specific food matrix. Moreover, the strong correlation between “synergistic effects” and various treatment terms points toward the rise of hurdle technology, where ultrasound is integrated with other nonthermal methods to enhance microbial lethality and sensory quality.

### 2.3. Cold Plasma Pretreatment

#### 2.3.1. Mechanism and Principles

CP was first designed for a wide range of applications in other industries, then emerged in the food sector with various applications [71]. As the fourth state of matter, plasma consists of ionized gases that contain electrons, free radicals, and ions [72]. Plasma can be classified into nonthermal (cold) and thermal plasma, which exist in either ground or excited states [73]. CP operates at near-ambient temperatures (30–60 °C), making it suitable for heat-sensitive food materials [74].

CP generates various reactive species including ROS such as ozone (O_3_), hydroxyl radicals (•OH), superoxide anions (O_2_•^−^), and reactive nitrogen species (RNS) such as nitrogen oxides and nitric oxide [75]. These reactive species interact with the food surface, causing chemical and physical modifications that can enhance drying efficiency and product quality [76,77].

Common CP generation systems in the food industry include DBD, atmospheric pressure plasma jet, corona discharge, and microwave plasma [78]. Among these, DBD is the most widely used for food applications due to its simplicity, scalability, and uniform treatment capability [79].

#### 2.3.2. Effects on Drying Performance

Deng et al. [80] studied jujube slices pretreated by CP for 15, 30, and 60 s on each side, followed by hot air drying at 50, 60, and 70 °C. Scanning electron microscopy investigation indicated that the application of CP significantly changed the surface topography of jujube slices by etching larger cavities, which can facilitate moisture transfer and consequently enhance drying rate and effective diffusivity.

In a study on apple slices, Li et al. [81] reported that CP pretreatment significantly modified the cellular structure of the apple slice surface by etching microchannels, which allowed rapid transfer of moisture during the drying process. These modifications improved the drying rate and decreased the drying time compared to control samples.

The enhancement in drying kinetics with CP pretreatment is attributed to several mechanisms: (1) etching and modification of the surface cuticle and wax layer, (2) creation of microchannels in the tissue, (3) partial degradation of cell wall components, and (4) modification of surface hydrophobicity [82,83,84].

#### 2.3.3. Quality Attributes

Deng et al. [80] found that CP pretreatment improved the contents of procyanidins, flavonoids, and phenolics by 53.81%, 33.89%, and 13.85% at most, respectively, and thereby enhanced antioxidant capacity by 36.85% at most. Besides, CP pretreatment reduced the production of HMF (a thermal degradation marker) by 52.19% at most.

Bao et al. [84] investigated CP pretreatment of blueberries and reported that the pretreatment improved the concentration of phenolic compounds (45.3 mg GAE g^−1^) and antioxidant compounds (799.8 µM ET) (*p* < 0.05). The CP pretreatment improved the preservation of bioactive compounds compared to conventional drying without pretreatment.

The mechanism behind the enhanced retention of bioactive compounds with CP pretreatment involves multiple factors: (1) reduced drying time and thermal exposure, (2) enzyme inactivation by reactive species, (3) modification of cell structure that may reduce enzymatic contact with substrates, and (4) potential stress-induced biosynthesis of protective compounds [85,86].

CP as a pretreatment is a novel and promising technique to inactivate enzymes and accelerate the drying rate of fruits and vegetables [87]. In addition to reducing drying time and increasing drying rate, CP pretreatment techniques can improve the dried products’ functional and nutritional quality attributes over conventionally pretreated and untreated samples [88,89].

Further, CP effectively disinfects microorganisms, inactivates enzymes and enhances nutritious qualities [90]. The antimicrobial effect of CP is particularly valuable for ensuring food safety in dried products, as it can achieve significant log reductions in bacteria, yeasts and molds without chemical residues [90,91]. The enzyme inactivation capability of CP has been demonstrated for polyphenol oxidase, peroxidase and lipoxygenase, which are responsible for quality deterioration during drying and storage [92,93].

CP is an emerging technology that uses ionized gas to decontaminate surfaces and modify tissue properties.

- Physical: CP treats the surface by “etching” the waxy cuticle of fruits and vegetables. This increases surface hydrophilicity, making it easier for moisture to escape during drying without damaging the internal structure.
- Chemical: It is highly effective at degrading pesticide residues and inactivating spoilage enzymes like polyphenol oxidase (PPO). However, ROS generated during treatment can cause localized lipid oxidation in high-fat produce.
- Sensory: One of the strongest advantages of CP is the preservation of “raw-like” sensory attributes. Since it operates at room temperature, the volatile flavor compounds remain intact, though high doses may cause a slight ozone-like off-odor in some cases.

Contemporary Scopus-indexed research suggests that CP is a chemically exclusive yet safe intervention. The plasma-induced nitrates and nitrites residues generated (10–60 mg kg^−1^) are statistically negligible compared to the endogenous nitrate levels in leafy vegetables (>2500 mg kg^−1^), thus posing minimal risk to the EFSA ADI of 0.07 mg kg^−1^ body weight while simultaneously offering the unique benefit of allergen and pesticide reduction [94,95].

#### 2.3.4. Energy Efficiency

CP pretreatment is an established, energy-efficient, non-thermal method for enhancing fruit and vegetable processing, primarily by improving the energy-intensive drying step [96]. The mechanism for these efficiency gains is surface modification. CP, an ionized gas, releases highly reactive species that cause microscopic etching and the formation of micro-fissures in the product’s waxy outer layers. This non-thermal damage significantly increases the effective moisture diffusivity (D_eff_), effectively opening up easier paths for water escape [97].

This enhanced permeability translates directly to substantial energy savings. CP pretreatment reduces drying time by 18% to over 50%), which in turn lowers the overall SEC of subsequent drying processes (e.g., hot-air) by approximately 25% to 46% [98,99]. The low electrical power required by CP systems (often 2–4 kWh ton^−1^ of food) further solidifies its status as a highly sustainable technology [97].

#### 2.3.5. Advantages and Disadvantages of CP Pretreatment

Table 3 shows the advantages and disadvantages of CP of fruits and vegetables before drying.

In this network, the size of each circle is a quantitative indicator of the keyword’s frequency, where dominant nodes such as “atmospheric cold plasma”, “microbial inactivation” and “food safety” identify the primary pillars of the field (Figure 3).

The aggregation of terms into clusters reveals a strategic partitioning of cold plasma research into approximately three to five major color groups. A primary red or blue cluster typically aggregates terms related to “microbial inactivation” and “decontamination”, focusing on the technology’s destructive nature toward foodborne pathogens and spoilage organisms. A secondary green or yellow cluster often focuses on “bioactive compounds” and “quality attributes”, reflecting research into how ROS and RNS interact with the food matrix to either preserve or enhance nutritional value. The presence of separate colors for “packaging” and “surface modification” indicates that cold plasma is also researched as a tool for developing active packaging materials that extend shelf life independently of direct food treatment.

As a hallmark nonthermal pretreatment, the diagram illustrates the significant co-occurrence of nodes such as “ROS and RNS”, “cell wall” and “extraction yield”. This represents the core scientific mechanism where cold plasma is used to disintegrate cellular structures, thereby liberating bioactive compounds without the use of heat or chemical solvents. The proximity of “phenolic content” and “antioxidant activity” to the central “cold plasma” node further emphasizes its application as a pretreatment to enhance the functional properties of fruits, vegetables, and agricultural and food biomass.

Other interrelationships and correlations are highlighted by “bridge” keywords such as “mechanism”, “stability” and “optimization”. These nodes sit at the intersection of various clusters, indicating that regardless of the specific application—whether it be “enzyme inactivation” in fresh-cut produce or “protein modification” in grains—the primary research goal remains the characterization of chemical and physical changes induced by plasma reactive species. The dense connectivity between “safety” and “organoleptic properties” reveals an increasing focus on ensuring that while microbial loads are reduced, the sensory quality (color, flavor, and texture) of the food remains uncompromised.

### 2.4. Osmotic Dehydration Pretreatment

#### 2.4.1. Mechanism and Principles

OD is a traditional pretreatment method that has gained renewed interest due to its combination with emerging technologies. To enhance drying efficiency, osmotic pretreatment has been introduced utilizing various osmotic solutes such as sucrose, glucose, fructose, salt, and alternative sweeteners [107,108]. Recent studies focus on optimizing pretreatment processes and minimizing drying time through the application of ultrasound waves in conjunction with osmotic dehydration [109].

US-assisted OD induces the formation of micropores in food structures, facilitates water removal, and enhances the penetration of osmotic solutes into the food matrix [110]. The osmotic process involves the immersion of food materials in hypertonic solutions, creating an osmotic pressure gradient that drives water removal from the product and solute uptake from the solution [111].

Key parameters affecting OD efficiency include solution concentration, temperature, immersion time, solution-to-sample ratio, agitation, and the type of osmotic agent used. These behaviors have been studied in various fruits where high concentrations generate higher osmotic pressure in the food tissue, accelerating mass transfer. Water loss is most pronounced during the first two hours of immersion [26,112].

#### 2.4.2. Effects on Drying Performance

Ramya and Jain [113] studied OD of kumquat slices before vacuum drying and found that OD decreased the total required drying time by up to 70 min compared to non-pretreated samples. The drying time was shortened as the initial moisture content decreased under the applied OD conditions. While the longest drying time was obtained by non-pretreated samples, OD samples at higher temperature and longer application time (OD/50 °C/90 min) showed the shortest drying time. The experimental results showed that drying time was reduced by increasing the temperature and application time of OD for kumquat slices. Moreover, OD treatment shortened the drying time between 20% and 70% compared to non-pretreated samples [113].

The weight and water loss results obtained from OD have a significant impact on the food drying industry, as it allows the removal of between 48% and 68% of water from the food in the first 3 h of the process, thus reducing conventional drying times, avoiding the exposure of food to high temperatures for long periods, and reducing nutrient losses due to high drying temperatures [111].

Utilizing OD as a pretreatment enables the acquisition of dehydrated fruits with a more authentic color profile. Numerous studies have documented a safeguarding impact on the color of fruits and vegetables when subjected to osmotic pretreatment. This color preservation is attributed to the protective effect of sugar infiltration, which can stabilize pigments and reduce enzymatic browning [114,115].

#### 2.4.3. Quality Attributes

Traditional osmotic agents like sucrose are being replaced or supplemented with alternative agents that offer additional benefits. The use of isomalto-oligosaccharide (IMO) as an osmotic agent for yellow peach slices. Notably, the highest total carotenoid content was found in dehydrated yellow peach slices pretreated by IMO-OD, followed by samples without OD, and samples with sucrose-OD pretreatment. In addition, the lowest water activity (0.517) was obtained in samples with IMO-OD for 5 h, which was beneficial for storage [116]. The assessment of water status and total carotenoid content of dehydrated yellow peach slices showed that IMO-OD pretreatment could better improve the quality of dehydrated fruits. Moreover, the use of IMO in OD treatment was a good alternative to sucrose [117].

Other alternative osmotic agents being explored include honey, maple syrup, fruit juice concentrates, glycerol, and polyols, each offering unique advantages in terms of health benefits, flavor profiles, and functional properties [118,119].

OD involves immersing produce in a hypertonic solution (usually sugar or salt) to draw out water via osmotic pressure.

- Physical: This process results in significant volume reduction and “structural collapse” as cells lose turgor pressure. It creates a dense, chewy texture rather than a crisp one.
- Chemical: There is a dual mass transfer: water moves out, while solutes (sugar/salt) move in. This can “fortify” the produce with preservatives or nutrients from the solution, but it also leads to the leaching of natural acids and minerals into the osmotic medium.
- Sensory: The sensory profile is heavily influenced by the solute. It enhances sweetness or saltiness and often improves color stability by reducing water activity (aw), which inhibits browning.

#### 2.4.4. Energy Efficiency

Unlike evaporative drying, which requires high latent heat to remove moisture (L_w_ = 2260 kJ kg^−1^ for water), OD facilitates water removal via a chemical potential gradient between the food matrix and a hypertonic solution [26]. The energy performance of OD is primarily dictated by the mass transfer rate and the management of the osmotic medium. Efficiency is maximized through the low operating temperatures (OD typically occurs between 30 °C and 50 °C, reducing the thermal load compared to traditional hot-air drying), reduced downstream load (by removing up to 50% of initial moisture before the final drying stage, OD reduces the total residence time in energy-intensive equipment like spray or freeze dryers), and solution management (the primary “energy cost” in OD is the regeneration of the osmotic solute. Utilizing membrane filtration or waste-heat evaporators to concentrate the spent syrup is essential for a favorable energy balance. Ultimately, while OD introduces a liquid-handling stage, the reduction in sensible and latent heat requirements during final dehydration often results in a net energy saving of 10% to 25% for the total process [120,121].

#### 2.4.5. Advantages and Disadvantages of Osmotic Dehydration Pretreatment

Table 4 shows the advantages and disadvantages of OD of fruits and vegetables before drying.

In this network, the size of each circle serves as a direct quantitative representation of a keyword’s frequency, where dominant nodes like “osmotic dehydration”, “mass transfer”, “kinetics” and “fruit” emerge as the primary anchors of the field (Figure 4). The thematic structure of the diagram highlights two primary research directions. First, the Mass Transfer and Kinetic Modeling cluster (red or blue) consolidates fundamental engineering concepts such as water loss, solute gain, diffusion, and mathematical modeling. This aggregation underscores a deep scientific commitment to deciphering the simultaneous counter-current exchange, where the food matrix is dehydrated as it absorbs solutes from the surrounding hypertonic medium. To complement this technical focus is the Quality and Bioactive Preservation cluster (green or yellow), which brings together attributes like antioxidant activity, total phenolic content, color, and texture. The grouping of these terms emphasizes the primary value proposition of osmotic dehydration: its capability as a low-temperature pretreatment to significantly reduce thermal degradation of sensitive pigments and nutrients, offering a superior alternative to the harsher conditions of traditional hot-air drying. As a hallmark nonthermal pretreatment, the diagram illustrates significant co-occurrence between “osmotic dehydration” and terms like “ultrasound”, “pulsed electric field” and “vacuum”. These nonthermal technologies are frequently researched as “assisted” methods to enhance cell membrane permeability, thereby accelerating dewatering and impregnation rates without the detrimental effects of high temperatures.

Other interrelationships and correlations are identified by “bridge” keywords such as “optimization” and “stability”, which sit at the intersection of various clusters. These nodes indicate that regardless of the specific food matrix—whether “apples”, “strawberries” or “carrots”—the overarching research goal is to optimize process parameters (temperature, time, and solute concentration) to achieve a stable, high-quality product. The dense connectivity between “osmotic dehydration” and subsequent drying steps like “freeze-drying” or “hot air drying” underscores its role as an energy-efficient upstream step that reduces the overall water load and improves the final texture and shelf life of the product.

### 2.5. High-Pressure Processing Pretreatment

#### 2.5.1. Mechanism and Principes

HHP or HPP could be assessed as an unconventional method to ensure the stability of food quality [130]. Pressure not only extends the shelf life of food by partly eliminating microorganisms but also changes its properties, providing the opportunity to create new food products with innovative structure. Technological advances in fruit processing create the possibility of combining traditional drying with unconventional methods of preservation [131].

HPP typically operates at pressures ranging from 100 to 600 MPa for food applications [132]. The high pressure causes compression of food materials and disruption of cell membranes through various mechanisms including protein denaturation, enzyme inactivation, and structural modification of plant tissues [133,134]. The pressure-induced changes in cell permeability facilitate moisture migration during subsequent drying.

#### 2.5.2. Effects on Drying Performance

It was observed that HPP pretreatment enhanced the drying rate and hence resulted in about 20% decrease in drying time for 300 MPa-treated samples compared to 4% for the 100 MPa-treated samples [135]. HP treatment reduced drying time by up to 20% compared to untreated samples. Generally, pressures of more than 100 MPa caused cell permeabilization, resulting in higher drying rates.

The enhancement in drying kinetics with HPP pretreatment is dose-dependent, with higher pressures generally producing more pronounced effects [136]. However, excessive pressure may lead to undesirable textural changes, necessitating optimization of treatment conditions [137].

#### 2.5.3. Quality Attributes

Pressure-treated samples had texture and color nearest that of the raw material compared to other pretreatments [138]. This advantage makes HPP pretreatment particularly attractive for high-value products where quality is paramount. On the other hand, HHP treatment resulted in incomplete rehydration when used alone, but this was improved when combined with freezing [139].

Initial drying rates were highest for water-blanched and frozen, pressure-treated and frozen, or just frozen samples, followed by hot-water-blanched and HHP-treated samples. The authors claimed that HHP treatment, in conjunction with subsequent freezing, can improve mass transfer in plant products and enhance product quality [140,141].

#### 2.5.4. Energy Efficiency

HPP pretreatment is a strategic “green” technology used to enhance the efficiency of subsequent food processes like drying, extraction, and cooking. While the HPP unit operation itself is energy-intensive, requiring significant electricity to power hydraulic pumps, it serves as a catalyst for substantial energy savings across the entire production line [142]. The core of this efficiency lies in the isostatic effect, which applies uniform pressure to the food’s cellular matrix. This process increases cell wall permeability and induces the formation of microchannels, which dramatically accelerates mass transfer [143].

When used before convective drying, HPP can reduce drying times by 20–40%. This significantly lowers the total energy consumed by industrial dehydrators, which are traditionally the most energy-heavy stages [144]. Although HPP’s direct electrical demand (roughly 2.5–3.2 kWh kg^−1^) is higher than simple thermal pasteurization, its ability to streamline energy-intensive downstream operations often results in a superior overall system energy balance. Unlike steam blanching, HPP is a NTP. It operates at ambient temperatures, eliminating the energy required for both the initial heating phase and the subsequent cooling loops needed for product stability. In “green” extraction workflows, HPP reduces the processing time and solvent volumes required to recover high-value bioactive compounds [145,146].

While HPP is characterized by high CAPEX and batch-operation constraints, its viability as a pretreatment for drying is contingent upon the synergistic reduction in drying energy (15–30%) and the creation of high-value, clean-label products. For bulk commodities, the cost-to-margin ratio remains prohibitive; however, for functional foods where bioactive retention is a primary price-driver, HPP serves as an economically justifiable alternative to chemical blanching [135,147].

HPP subject food to extreme pressure (up to 600 MPa), which affects large molecules like proteins and starches while leaving small flavor molecules untouched.

- Physical: High pressure causes instantaneous and uniform compression. This can lead to starch gelatinization and protein denaturation, often resulting in a firmer, more “cooked” texture compared to raw produce, but fresher than heat-sterilized produce.
- Chemical: It effectively inactivates microorganisms and enzymes without the need for heat, preserving the nutritional bioactives and the primary structure of vitamins.
- Sensory: HPP is the “gold standard” for preserving the original flavor and color of juices and fresh-cuts. The sensory experience is nearly identical to the fresh counterpart, though the altered texture may be noticeable in high-starch vegetables.

#### 2.5.5. Advantages and Disadvantages of HPP

Table 5 shows the advantages and disadvantages of HPP of fruits and vegetables before drying.

The co-occurrence network diagram for HPP within the food technology domain reveals a highly integrated research landscape centered on microbial safety, quality preservation, and biochemical analysis (Figure 5). In this visualization, the size of each circle is a direct quantitative indicator of a keyword’s frequency or research weight, where dominant “anchor” nodes such as “inactivation”, “*Listeria monocytogenes*” and “*Escherichia coli*” emerge as the primary pillars of the field. These large nodes are surrounded by a network of smaller circles, such as “nisen”, “salmonella”, and “carotenoid”, which represent more specialized or emerging sub-topics. The correspondence between circle size and its specific color defines a thematic hierarchy; while the color categorizes research into distinct scientific sub-domains, the node size signifies which concepts serve as the foundational hubs for those particular disciplines.

The aggregation of terms into clusters reveals a strategic partitioning of research into several major color groups. A prominent red cluster acts as the primary hub for food safety and microbiology, aggregating terms like “inactivation”, “packaging” and specific pathogens, illustrating the technology’s maturity as a preservation tool for products like “beef” and “fillet”. In contrast, the green cluster shifts toward structural and enzymatic studies, focusing on “microstructure”, “enzyme activity” and “processing technology”. The blue and purple clusters on the right of the map represent the analytical and developmental frontiers, where keywords like “analysis”, “determination”, “mass spectrometry” and “liquid chromatography” indicate a strong focus on characterizing the chemical constituents and metabolites resulting from pressure-induced changes.

As a hallmark nonthermal pretreatment, the diagram illustrates a significant co-occurrence between “inactivation” and terms related to nutritional quality, such as “bioaccessibility” and “phenolic”. Scientifically, this represents the core advantage of HPP: achieving high microbial lethality while preserving heat-sensitive bioactive compounds. The proximity of “carrot juice” and “food matrix” to the inactivation hub underscores ultrasound’s role as a pretreatment that maintains the “fresh-like” characteristics of liquid and solid foods without the detrimental effects of thermal pasteurization.

Other interrelationships and correlations are identified by “bridge” keywords such as “characterization”, “development” and “modeling”, which sit at the intersection of various clusters. These nodes indicate that, regardless of the specific food application, the overarching research goal is to characterize the “structural change” and “quality characteristic” of the product to ensure stability. Furthermore, the dense connectivity between “inactivation” and “quality attribute” suggests an increasing focus on the “hurdle technology” concept, where HPP is optimized to balance safety with the retention of sensory and nutritional profiles.

### 2.6. Freeze-Thaw Pretreatment

#### 2.6.1. Mechanism and Principes

FT is a non-thermal physical pretreatment consisting of two steps: freezing material to its freezing point and thawing the frozen material at higher temperatures. FT pretreatment is an efficient method to improve the drying rate by changing the cell membrane’s permeability and destroying the cell wall’s structure [154]. It has been proven that FT pretreatment can significantly improve the performance of different thermal drying processes owing to the rupture of cell membranes and walls, preventing structural deformation during drying [155].

FT treatment is a promising pretreatment method that uses ice crystals formed during the freezing process to destroy plant tissues and cell membranes, thereby increasing drying efficiency, reducing energy consumption, or improving yield of products [156]. The size, shape, and location of ice crystals depend on the freezing rate, with rapid freezing producing smaller intracellular crystals and slow freezing producing larger extracellular crystals [157,158].

#### 2.6.2. Effects on Drying Performance

Wang et al. investigated FT pretreatment of carrot slices and found that it improved the drying rate (from 0.0188 to 0.0261 min^−1^), reduced the total energy consumption (from 4.77 to 3.73 kWh kg^−1^), and resulted in a more porous structure of the final product with low shrinkage rate (from 83.39 to 73.04%), reduced hardness (from 41.67 to 24.49 N), and crisp texture (from 0.36 to 0.17 s) [159]. Cell membranes were destroyed and microchannels formed after pretreatment.

Dalmau et al. [160] studied the effect of LNIon various berries. Liquid nitrogen pretreatment reduced epicarp thickness for all berries, with maximal thickness reduction (47%) for blueberries after five cycles of LNI, mainly due to the dewaxing of the berry surface observed by SEC. The freeze-drying times for these berries were shorter than for other drying methods under study. The drying constant k_2_ increased markedly with LNI pretreatment for all drying methods, but especially for freeze drying, for which it increased 1.5 to 6.5 times for different fruits.

#### 2.6.3. Quality Attributes

The formation of ice crystals (microstructural integrity) causes mechanical stress that ruptures cell walls, creating a porous network. This increased porosity is a defining attribute, as it facilitates faster moisture migration [161]. Due to the structural damage, FT-treated materials exhibit significantly reduced drying times and lower energy consumption during dehydration processes like vacuum freeze-drying [162].

FT can enhance the extractability of bioactive compounds (e.g., phenolics and flavonoids) by breaking down cellular barriers, though extremely slow freezing may lead to some nutrient degradation. A common result is textural softening, characterized by reduced hardness and chewiness. While this aids in producing “crispy” snacks, it also improves the rehydration capacity of the final dried product [163].

FT cycles use the formation of ice crystals to mechanically rupture the internal cellular framework.

- Physical: Large ice crystals act as “needles” that puncture cell walls. Upon thawing, the tissue becomes very soft and highly permeable. This results in the fastest drying rates but often leads to significant structural collapse and “shriveling”.
- Chemical: The cell rupture allows enzymes and substrates to mix, which can accelerate enzymatic browning if the produce is not blanched or chemically treated immediately after thawing.
- Sensory: The texture is usually the most compromised in FT, often becoming mushy. However, for products intended for purees or as ingredients in baked goods, the concentrated flavor resulting from high moisture loss is a benefit.

#### 2.6.4. Energy Efficiency

FT pretreatment significantly enhances the energy efficiency of subsequent dehydration processes by reducing the internal resistance to moisture migration. The core mechanism involves the formation of large ice crystals that physically rupture cell walls and membranes, creating a network of micropores and channels [164].

This structural modification facilitates faster mass transfer, which can reduce total drying time by 20% to 60% depending on the material. Because drying—particularly vacuum freeze-drying—is one of the most energy-intensive operations in food processing, shortening the cycle directly leads to substantial savings. For instance, studies on products such as lotus root and okra have shown total energy consumption reductions of approximately 17% to 35% (measured in kWh kg^−1^), even when accounting for the energy required for the initial freezing stage [165,166]. The efficacy FT pretreatment is highly commodity-dependent. While it is a superior strategy for overcoming the waxy cuticle of whole berries and the dense matrix of root vegetables, it is contraindicated for high-sugar soft fruits and leafy greens due to irreversible structural collapse and significant drip loss. For these sensitive matrices, dehydro-freezing or fast-freezing rates must be employed to mitigate quality degradation [162,167].

#### 2.6.5. Advantages and Disadvantages of Freeze-Thaw Pretreatment

Table 6 shows the advantages and disadvantages of FT of fruits and vegetables before drying.

The thematic landscape of FT research is defined by three primary scientific pillars (Figure 6). First, the Dehydration and Mass Transfer cluster (red or blue) centers on water loss, diffusivity, and shrinkage, reflecting the industrial use of FT to accelerate moisture migration through the creation of porous structures. This is complemented by the Quality and Bioactive cluster (green or yellow), which groups attributes like antioxidant activity and phenolic content to highlight FT’s efficacy in preserving heat-sensitive nutrients compared to traditional thermal methods. Finally, the Structural and Physical cluster focuses on microstructure, cell wall damage, and porosity, identifying the mechanical formation of ice crystals as the fundamental mechanism that dictates the final food texture and extraction efficiency.

As a leading nonthermal pretreatment, the diagram illustrates significant keyword co-occurrence between “freeze-thaw” and other emerging technologies like “ultrasound” and “microwave.” These co-occurrences represent hybrid pretreatment strategies where ultrasound or microwaves are used to assist the thawing phase to minimize structural damage and enzymatic browning. The dense connectivity between “microstructural damage” and “extraction yield” underscores the core scientific mechanism of FT: utilizing thermal shock and ice crystallization to disintegrate cell walls, which facilitates the subsequent release of bioactive compounds or oil without the use of chemical solvents or high heat.

Other interrelationships and correlations are identified by “bridge” keywords such as “modeling” and “optimization”, which sit at the intersection of various clusters. These nodes indicate that regardless of the food matrix—ranging from “fruits and vegetables” to “seeds”—the overarching research goal is to optimize the number of cycles and freezing/thawing rates to balance drying efficiency with the retention of functional properties. Furthermore, the correlation between “rehydration ratio” and “porous structure” reveals that FT is uniquely valued for producing dried products with superior structural integrity and faster preparation times.

The unique network visualization (Figure 7) of keyword co-occurrence for various pretreatments in food technology illustrates a complex ecosystem where the Quality Attributes node occupies a central position, acting as the primary hub connecting physicochemical changes with process parameters. The size of each circle (node) in the diagram directly reflects the frequency of the specific term’s appearance, with the largest circles—such as pretreatment methods—identified as key research pillars that generate the densest interaction networks.

The aggregation of terms within these clusters clearly differentiates technological approaches: the red clusters are tightly linked to mass transfer through Osmotic Dehydration and High Processing Dehydration, while the dark-green cluster at the right, focused on High Freeze-Thaw Cycling, emphasizes the structural integrity of food during thermal cycles. The number of colors within each cluster indicates the heterogeneity of the research field; for instance, the red yellow/blue cluster encompassing Ultra Sound, Extraction and Determination demonstrates a high degree of integration across various sub-disciplines focused quantification of bioactive compounds like “polysaccharides” and “flavonoids”.

In the context of NTP, the diagram illustrates intense keyword co-occurrence among terms such as OD, US and HPP, which are mapped in close proximity to nodes for bioactive compound extraction. Particularly significant are the interrelationships based on correlations between clusters, where the thickness of the lines (links) between Quality Attributes and specific pretreatments suggests that the preservation of sensory and nutritional properties is the primary driver of innovation in the non-thermal sector. These correlations also reveal “bridges” between fundamental food science and industrial application, linking laboratory terms like Quality Attributes to commercially relevant outcomes maintaining the integrity of hydrocolloids like pectin and starch.

## 3. Combined Pretreatment Strategies

### 3.1. PEF Combined with OD

Wiktor et al. [173] demonstrated that PEF and OD pretreatment can accelerate the time-consuming drying process and minimize its high energy demands. The effect of PEF and OD preprocessing conditions and OD composition on mass transfer kinetics (water loss, solid gain, water activity) and quality properties (color, texture, total sensory quality) during OD and AD of pumpkin was studied. Application of PEF (2.0 kV cm^−1^–1500 pulses) significantly enhanced mass transfer during AD (increased effective diffusivity coefficient D_eff_ and DR, respectively). PEF and OD treatments led to a significant reduction of processing time by 12% and 10%, respectively (*p* < 0.05). The maximum reduction of processing time by 27% (*p* < 0.05) compared to untreated samples resulted from the combined use of PEF and OD as pretreatments prior to AD. When PEF pretreatment was combined with OD prior to AD, the corresponding energy consumption was 50% less than the respective energy required for non-processed samples. This dramatic reduction in energy consumption demonstrates the synergistic effect of combining these two pretreatment technologies [174,175]. Generally speaking, the PEF + OD combination can lead to a 20% to 30% reduction in total energy consumption and reduce final drying time by 40% to 60%.

The mechanism behind the synergistic effect involves PEF-induced membrane permeabilization, which enhances the osmotic mass transfer during OD, and the combined effect of reduced initial moisture content (from OD) and increased tissue permeability (from PEF) during the final air-drying step [176].

The integration of PEF with OD is primarily designed to accelerate mass transfer by opening cellular “gates” before immersion.

- Physical: PEF creates permanent nanopores in the cell membranes (electroporation), which allows the osmotic solution to penetrate the tissue much more deeply and rapidly. This reduces the total processing time needed to reach a target moisture level, effectively minimizing the structural collapse often seen in long-term soaking.
- Chemical: This synergy enhances the “solid gain” of beneficial solutes (like calcium or antioxidants) from the osmotic solution into the fruit matrix. Because the process is non-thermal, it prevents the degradation of heat-sensitive pigments and vitamins, though some water-soluble minerals may leach out through the PEF-induced pores.
- Sensory: The resulting product typically maintains a better balance between sweetness and acidity. The texture is firmer than produce subjected to OD alone, as the shortened immersion time prevents the tissue from becoming overly “waterlogged” or mushy.

### 3.2. US Combined with OS Dehydration

Wang et al. [177] investigated the novel use of US pretreatment (10, 20, and 30 min) assisted with OD to improve drying efficiency and product quality of goji berries. The results showed that goji berries treated with US-assisted osmotic dehydration lost more water and gained more solids during OD than goji berries treated with OD only. Furthermore, the US-assisted OD method shortened the drying time for FT goji berries and lowered the water activity of the dried goji berries. This may be due to the destruction of the wax layer on the surface of the goji berries by US. The rehydration rate of the goji berries treated with US for 20 min was significantly higher than that of the samples dried by OD only [177].

Yan et al. [178] studied pineapple slices and found that OD and US pretreatments enhance microwave-assisted vacuum freeze-drying efficiency, reducing drying time by up to 35% and energy consumption by 30%. The FT process showed no significant difference in moisture curves between pretreated and untreated samples, but the pretreated samples had lower initial moisture content, leading to a potential 35.01% reduction in drying time. The study also evaluated the quality of the dried samples using eight performance indicators, finding that OD pretreatment improved sugar content, crispiness, and flavor, whereas ultrasonic pretreatment enhanced rehydration rate, reduced final moisture and sugar content, and resulted in softer texture. Additionally, US pretreatment with 120 W power and 40 min duration significantly reduced drying time and energy consumption by up to 30.02% [178]. US + OD typically reduces total drying time by 18% to 45% and 15% to 25% reduction in total process energy.

This combination uses acoustic energy to stir the osmotic boundary layer and micro-scrub the surface of the produce.

- Physical: Ultrasound creates microscopic “channels” through cavitation. These channels act as highways for water to leave the fruit and for solutes to enter. This combination is particularly effective for produce with thick skins (like berries or grapes) that normally resist osmotic flow.
- Chemical: The mechanical action of ultrasound can help in the “debinding” of polyphenols from the cell wall, often leading to a higher concentration of bioavailable antioxidants in the final product. However, prolonged exposure to ultrasound in a liquid medium can lead to some oxidation of fats or pigments due to the formation of free radicals.
- Sensory: The sensory profile is characterized by excellent color retention. The cavitation effect inhibits surface browning by displacing oxygen, leading to a vibrant, “fresh” appearance. The mouthfeel is usually described as porous and slightly crunchy.

### 3.3. PEF and US with Drying

Llavata et al. [156] assessed the influence of FT and PEF pretreatments in conventional and airborne US-assisted drying (50 °C) of orange peels. None of these pretreatments alone managed to reduce processing times significantly, but when combined with US-assisted drying, they produced a significant shortening of the process. This was particularly important in the lower intensity PEF pretreatment tested (0.33 kJ kg^−1^), indicating the existence of optimum conditions to carry out the pretreatments. Applying PEF pretreatment at 1.0–5.0 kV cm^−1^ (Z = 0.1–0.8) to orange peel waste significantly improved drying efficiency at temperatures below 55 °C, increasing moisture diffusivity by up to 25% and reducing energy consumption by up to 15 MJ kg^−1^ without altering antioxidant activity [179].

Llavata et al. [180] assessed the combined influence of drying temperature (40–70 °C), PEF pretreatment (0–5000 V), and ultrasound (US; 0–50 W) application during the drying of kiwifruit. The increase in temperature, the use of PEF pretreatment, or the US application during drying accelerated the process, but it was the combination of the three that led to the highest values of the effective diffusivity and the mass transfer coefficient. Over the range of different conditions studied, the functional and antioxidant properties of dried kiwifruit were not significantly affected, while the color presented some differences, mainly related to the color (L* and a* coordinates) [180]. This process typically reduces total drying time by 40% to 60% and 30% to 45% in energy savings.

The synergistic effects observed in these studies highlight the potential of multi-level intervention strategies that act on different mechanisms simultaneously. PEF primarily affects cell membrane permeability, while US creates mechanical effects through cavitation, and their combination provides complementary pathways for enhanced mass transfer [181].

This multi-stage approach is one of the most advanced “green” processing methods, focusing on maximum water removal efficiency and structural preservation.

- Physical: By combining PEF (internal membrane disruption) and US (surface and channel creation), the internal resistance to moisture movement is almost entirely neutralized. When followed by hot air or vacuum drying, the moisture evaporates with minimal energy input, preventing the “case hardening” (hard outer shell) often found in traditionally dried fruits.
- Chemical: This sequence allows for significantly lower drying temperatures. As a result, the retention of volatile flavor compounds and thermolabile nutrients (like carotenoids) is remarkably high. The rapid drying also limits the time available for enzymatic browning to occur.
- Sensory: These products often have a unique “puffed” or airy texture rather than being leathery. The flavor is intense and concentrated, and the rehydration properties are superior, meaning they return to a more natural state when used in soups or cereals.

### 3.4. US Combined with Chemical Pretreatments

Zang et al. [182] investigated the combination of US with chemical pretreatments for sweet potato slices subjected to radio-frequency vacuum drying. The study found that combinations of pretreatment technologies improved drying rate by 10.34–41.38%. US-CMC) pretreatment was the best combination for improving quality properties. US-CMC combined with radio-frequency vacuum drying exhibited the highest overall acceptance and better texture. US combined with chemical pretreatment reduced energy consumption by 3.22–19.34% [182] and 40–60% in drying time reduction.

The combination of US with edible coating materials such as CMC provides dual benefits: the US treatment modifies the tissue structure to enhance mass transfer, while the coating provides a protective barrier that helps retain bioactive compounds and improves the final texture of the dried product [183].

If the goal is pure industrial throughput, the combination of PEF + US is the undisputed winner. It removes both internal (cellular) and external (boundary layer) resistance. It is the only method that consistently cuts drying time by more than half, though it carries the highest equipment setup cost. For premium snacks where color, vitamins, and “fresh” smell are the priority, US + OD is the gold standard. The ultrasound waves perform a “micro-cleaning” of the surface and prevent the browning enzymes from taking hold, while the OD step infuses natural sugars or flavors. PEF is unique because it modifies the texture. Because it “leaks” the cells without destroying the cell wall, it is excellent for products that need to be rehydrated later. It produces a “softer” internal structure that absorbs water faster than the “rubbery” texture often found in conventional dried foods. This remains a niche but necessary method. No amount of PEF or US alone can efficiently dry a whole grape or blueberry because of the waxy cuticle. The chemical pretreatment “cracks” the armor, and the ultrasound ensures the drying begins immediately, preventing the fruit from fermenting or spoiling during a long drying cycle.

This strategy uses ultrasound to “inject” or deeply infuse chemical agents (such as citric acid, calcium chloride, or anti-browning agents) into the produce.

- Physical: The ultrasound-induced cavitation acts as a mechanical pump, forcing the chemical solution into the intercellular spaces far more effectively than simple dipping. This ensures that the protective chemicals reach the core of the fresh-cut piece, not just the surface.
- Chemical: The primary goal here is the stabilization of the chemical matrix. Calcium ions can be driven into the cell wall to cross-link with pectin, significantly strengthening the “skeletal” structure of the vegetable. This prevents the enzymatic breakdown that leads to spoilage.
- Sensory: This combination is the gold standard for maintaining “crispness” and “whiteness” in products like sliced apples or potatoes. By deep-infusing anti-browning agents, the produce maintains its aesthetic appeal for a much longer shelf-life without the metallic or chemical aftertaste sometimes associated with heavy surface sprays.

### 3.5. Multiple Pretreatment Combinations

Several researchers have explored the combination of three or more pretreatment methods to achieve maximum benefits. For example, the combination of blanching, OD and US has been investigated for various fruits and vegetables [184,185]. These triple combinations can provide enzyme inactivation (from blanching), moisture reduction and sugar infusion (from OD), and enhanced mass transfer (from US) [186].

Similarly, the combination of FT with PEF or US has shown promising results, as the FT cycle creates initial structural damage that enhances the effectiveness of subsequent electrical or mechanical treatments [187,188].

Generally speaking, the new and combined technologies combining NTP with combined drying have been investigated, and some studies point out that the combined treatment techniques of two or more pretreatment methods, such as PEF and US, can further improve the drying characteristics of fruits and vegetables [63,189,190,191,192]. Since PEF pretreatment affects the structure of the product, while the treatment effect of US depends on the internal structure of the product, the combination of both can have a synergistic effect. The study of the interaction or synergy of several pretreatment or drying methods may lead to a greater breakthrough in further improving drying efficiency and product quality.

The synergistic mechanisms can be explained through several complementary pathways:

1. Sequential structural modification: Different pretreatments act on different structural components (cell membranes, cell walls, cuticle layers), and their sequential application creates cumulative effects.
2. Enhanced accessibility: Initial pretreatments may enhance the accessibility and effectiveness of subsequent treatments by modifying tissue permeability.
3. Complementary mechanisms: Physical treatments (PEF, US, FT) primarily affect tissue structure, while OD treatments modify the compositional gradient, and their combination addresses both structural and driving force aspects of mass transfer.
4. Reduced treatment intensity: Combined pretreatments may allow for reduced intensity of individual treatments, potentially minimizing undesirable side effects while maintaining or enhancing overall effectiveness.

**Figure 8:**
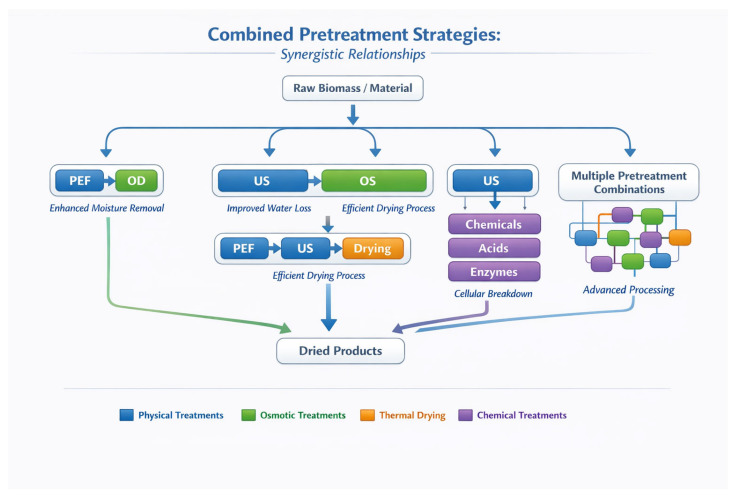
Schematic representation of synergistic non-thermal pretreatment strategies for fresh-cut produce.

The diagram (Figure 8) illustrates the integration of physical treatments of combined pretreatments to optimize mass transfer, reduce drying time, and enhance the quality of the final dried product. The evolution of the fresh-cut drying industry increasingly relies on the strategic coupling of non-thermal technologies to overcome the limitations of individual processing steps. The integration of PEF with OD, for instance, utilizes the mechanism of electroporation to significantly enhance cellular permeability. By creating a more “porous” tissue structure, this synergy allows for a reduction in osmotic immersion times, which is critical for mitigating microbial fermentation risks in high-moisture sliced produce. Furthermore, it enables a more precise control over solid gain, ensuring that moisture is removed without oversaturating the fruit with solutes, thereby maintaining the product’s original flavor profile. The combination of US and OD exploits the mechanical advantages of acoustic cavitation and the “sponge effect.” The rapid cycles of compression and expansion induced by ultrasound create microscopic channels within the vegetable matrix. These pathways facilitate more efficient water loss and remain open during subsequent processing, effectively preventing the phenomenon of case hardening—a common defect in sliced products where a rigid exterior shell forms and traps internal moisture. Additionally, the mechanical action of US serves a dual purpose by aiding in surface decontamination during the dehydration phase.

When these technologies are organized into a sequential PEF-US drying framework, the result is a significant peak in thermodynamic efficiency. This approach addresses a long-standing scientific gap in pre-drying preparation by targeting multiple structural layers simultaneously: PEF destabilizes the lipid bilayer of the cell membrane, while US disrupts the cell wall and the broader tissue matrix. This systemic reduction in mass transfer resistance is typically reflected in the highest recorded values of D_eff_, offering a robust rationale for the industrial adoption of non-thermal sequences to lower energy footprints.

Finally, the synergy between US and chemical or enzymatic pretreatments is paramount for rigorous quality assessment. The application of US enhances the mass diffusivity of anti-browning agents and pH regulators, ensuring they penetrate the core of the produce slices more uniformly than conventional dipping methods. Because these interactions occur under non-thermal conditions, the thermal degradation index remains remarkably low. This preservation of the food matrix ensures that thermolabile bioactives, natural antioxidants, and the vivid chroma of the fresh-cut produce are retained in the final dried product, meeting the rising consumer demand for “clean-label” and nutrient-dense ready-to-eat snacks.

Table 7 summarizes the various pretreatment technologies for fresh-cut fruits and vegetables, highlighting their primary mechanisms, impacts on quality, and current industrial standing.

When a product is “fresh-cut”, the mechanical injury ruptures cells at the surface, leading to:

- Enzymatic browning: PPO and POD met phenolic substrates
- Cellular leakage: Loss of turgor pressure and leaching of intracellular nutrients (sugars, vitamins), creating a sticky surface that can hinder airflow.

## 4. Advancements in Non-Thermal Pretreatment for the Fresh-Cut Produce Drying Industry: Market Trends, Quality Determinants, and Technological Frontiers

The global demand for convenience and healthy snacking has catalyzed the rapid growth of the fresh-cut fruit and vegetable industry. Fresh-cut products, defined as those physically altered (peeled, sliced, or diced) while remaining in a fresh state, represent a burgeoning segment of the processed food market. However, the inherent high moisture content and physical wounding associated with slicing make these products extremely perishable. Drying serves as a critical preservation strategy, but the industrial transition from whole-fruit to fresh-cut drying has exposed significant technical hurdles. Traditional thermal methods, while cost-effective, often lead to severe quality degradation. Consequently, NTP technologies have emerged as an essential prerequisite for maintaining the “fresh-like” attributes that modern consumers demand [193,194].

The economic footprint of the fruit and vegetable processing sector is expanding at a significant rate [193,195,196]:

- Market size: As of 2026, the global fruit and vegetable processing market is valued at approximately $276.82 billion, with a projected compound annual growth rate (CAGR) of 8.2% leading into 2030.
- Segment growth: The dried and dehydrated segment is one of the fastest-growing categories, fueled by the HoReCa sector and a 7.52% growth rate in RTE convenience snacks.
- Waste mitigation: Industry reports indicate that 25–30% of global food waste occurs during processing. Efficient drying of fresh-cuts is identified as a primary solution to valorize specific produce and surplus yield into shelf-stable, high-value products.

Freshly sliced products are biologically distinct from intact commodities. The physical act of slicing triggers physiological responses that must be addressed before the drying front is applied [194,197]:

1. Tissue wounding and respiration: Slicing increases the respiration rate and ethylene production, accelerating the metabolic breakdown of the product.
2. Oxidative browning: The rupture of cell walls allows PPO to come into contact with phenolic substrates, causing rapid discoloration (browning) at the cut surfaces.
3. High surface area: While an increased surface area facilitates faster moisture removal, it simultaneously increases exposure to oxygen and light, leading to the rapid degradation of heat-sensitive bioactives like Vitamin C and anthocyanins.

NTP technologies act as “enablers” for the drying process by modifying the food matrix without the use of heat. The rationale for adopting NTP lies in the limitations of traditional pre-drying methods, such as hot-water blanching. Traditional blanching causes the loss of water-soluble nutrients. Research shows that NTP avoids this “wash-out” effect, maintaining the nutritional integrity of the slice [198]. Thermal pretreatments often cause “thermal softening” via pectin degradation. NTP (like PEF or HHP) maintains the turgor pressure of the cells, which is vital for the “crispness” of the final dried product [199].

To compare TP vs. NTP processing, the following metrics are utilized [200]:

1. DR: The speed of moisture removal per unit of time.
2. RR: A measure of the dried slice’s ability to regain its original structure.
3. SEC: The total energy required to remove 1 kg of water. NTP often reduces by increasing the permeability of the tissue.

The most significant recent advancements involve hybrid systems and smart monitoring, VPD, EPD and ALI. VPD is a novel method where the chamber cycles between vacuum and atmospheric pressure, creating a “pull-push” effect on internal moisture, significantly reducing internal resistance to water migration [201]. EPD uses as a finishing step after NTP to create an airy, porous texture in fruit chips, mimicking the quality of freeze-drying at a fraction of the cost [202]. ALI is a real-time monitoring of browning via hyperspectral imaging allows for automated adjustment of NTP intensity during the production run [193].

## 5. Conclusions

Non-Thermal Pretreatment (NTP) technologies are transformative tools for the fresh-cut industry, bridging the gap between processing efficiency and the high-quality standards demanded by modern consumers. This review highlights that while traditional methods like OD remain industrially dominant, emerging NTP—specifically PEF, US, and HPP—offer superior retention of bioactives (up to 95%) and significant energy reductions (up to 50%). The synergy found in combined strategies, such as PEF-OD and US-OD, represents a critical shift toward “hurdle technology” that optimizes mass transfer without the structural damage typical of thermal methods.

Key findings and conclusions include:

1. Effectiveness of NTP: NTP technologies have demonstrated significant potential for reducing drying time (20–55%), enhancing mass transfer, and improving quality attributes while maintaining or enhancing nutritional content. These methods address consumer demands for minimally processed, high-quality products.
2. Synergistic effects of combined pretreatments: The combination of two or more pretreatment methods (e.g., PEF + OD, US + OD) produces synergistic effects that exceed the benefits of individual treatments. Energy savings of up to 50% and drying time reductions of up to 35% have been reported with combined pretreatments.
3. Mechanisms of action: Pretreatments enhance drying through multiple mechanisms including cell membrane electroporation, cavitation-induced tissue disruption, ice crystal formation, osmotic pressure gradients, and plasma-induced surface modification. Understanding these mechanisms enables rational selection and optimization of pretreatment methods for specific products and applications.
4. Quality preservation: Appropriate pretreatments significantly improve the retention of bioactive compounds (vitamins, polyphenols, carotenoids), preserve color and sensory attributes, and enhance rehydration properties. Bioactive compound retention can be improved by 30–95% compared to conventional drying without pretreatment.
5. Energy efficiency and sustainability: Pretreatments contribute to sustainability by reducing energy consumption, minimizing water usage (for non-thermal methods), eliminating chemical residues, and reducing food waste through improved product quality. The energy invested in pretreatment is typically much lower than the energy saved during subsequent drying.
6. Industrial application challenges: While many pretreatment technologies have been extensively studied at laboratory scale, industrial implementation faces challenges related to equipment scale-up, treatment uniformity, process control, and economic viability. However, the growing market for high-quality dried products and increasing energy costs provide strong economic incentives for adoption.
  - TRL 9 (Commercial): OD is widely used industrially, particularly for tropical fruits.
  - TRL 8–9 (Commercial/Safety focus): HPP is commercially established for safety, though its specific application as a drying pretreatment remains limited due to high capital investment.
  - TRL 6–7 (Demonstration/Pilot): PEF and US are in the demonstration phase, with growing interest for drying applications despite challenges in field uniformity and equipment scale-up.
  - TRL 5–6 (Pilot/Specialized): FT is effective for specific textures, such as carrot slices, but faces niche adoption.
  - TRL 4–5 (Lab/Experimental): CP remains largely experimental for drying, facing challenges in treatment uniformity and equipment design.
7. Future directions: Emerging trends include the development of hybrid pretreatment systems, integration of digital technologies for process monitoring and control, exploration of novel technologies (pulsed light, supercritical CO_2_), and increased focus on sustainability through renewable energy integration and circular economy approaches.
8. Product-specific optimization: The optimal pretreatment strategy varies depending on product characteristics (tissue structure, bioactive compound profile), desired quality attributes, and economic considerations. Multi-objective optimization approaches are needed to balance drying efficiency, quality preservation, and economic viability.

In summary, the transition toward non-thermal, synergistic pretreatments is no longer a matter of feasibility, but of economic and technical refinement for industrial-scale deployment.

## Figures and Tables

**Table 1:** The advantages and disadvantages of PEF pretreatment of fruits and vegetables before drying.

Category	Advantages	Disadvantages	References
Drying Time	Significantly reduced drying time (by more 15–35%)	Potential for Nutrient degradation/loss (Vitamin C, phenolics)	[18,34,35,36,37,38,39,40]
Energy Efficiency	Reduced energy consumption (low energy input 2–20 kJ kg^−1^) Energy saving 10–20%	Current PEF devices are not efficient for industrial application	
Quality—Color	Retention of colors (preserving color)	Potential for undesirable color change (enzymatic reactions PPO)	
Quality—Texture	Higher porosity More uniform shape, better retention of volume, and crispier	Ambiguous effect on texture (firmness): PEF can increase or decrease firmness depending on the tissue and specific PEF parameters	
Nutrient Retention	Improves the retention rate of nutrients	Potential loss of sugars, vitamin C	
Antioxidant Activity	Enhanced retention of bioactive compounds	Significant decrease in antioxidant activity in some air-dried fruits and vegetables	
Rehydration	Improved final product quality in terms of rehydration capacity and reduced shrinkage	Significant loss of cell viability (irreversible electroporation, for PEF at 250 or 400 V cm^−1^)	
Mechanism	Improved mass transfer during subsequent processes (osmotic dehydration)	PEF parameters must be well-chosen to obtain desired outcomes; browning reduction mechanisms need more study	
Cost—Equipment	Commercial-scale costs approximately 1 Euro ton^−1^ for cell disintegration applications (Pulsemaster) Compact design, continuous operation capability, and high energy efficiency for membrane permeabilization	Mentions high machinery cost and dependence on medium composition as drawbacks	
Safety	Maintaining physicochemical properties of dried products	High initial capital cost for the PEF equipment	
Tissue Damage	PEF-treated apple tissue exhibited higher crunchiness and brittleness	Limited inactivation of microorganisms (food safety)	

**Table 2:** The advantages and disadvantages of US pretreatment of fruits and vegetables before drying.

Category	Advantages	Disadvantages	References
Drying Time	US pretreatment, combined with ultrasound-assisted air drying and other techniques, reduced processing time by 20–40%	Limited effectiveness for certain products compared to other pretreatment methods; Initial high investment host for the equipment	[61,62,63,64,65,66,67,68]
Energy Efficiency	US pretreatment considerably decreases energy consumption for most fruits tested Energy saving 15–25%	Current ultrasonic devices are not efficient for industrial application	
Quality—Color	US treatments can significantly improve the color of dried fruit and vegetable products	High-frequency US cleaning damages the outer layer of fruits and vegetables tissue and causes nutrient loss	
Quality—Texture	US pretreatment could reduce hardness of dried products Improves firmness	Excessive cavitation can cause tissue damage	
Nutrient Retention	US treatment reduces water activity, enhances product color, and decreases loss of nutrients including flavonoid content, antioxidant activity, vitamin C and total phenolic content	Nutrient losses in medium and impedance mismatch are main limitations	
Antioxidant Activity	High power US (≥300 W) increases total phenol content and antioxidant activity, with short-duration ultrasound (t < 15 min) increasing antioxidant activity	Parameter selection must be precise to avoid negative effects	
Rehydration	Shrinkage and water activity can be minimized using pretreatments like US (improving the rehydration ratio)	US pretreatment tends to remove sugars, vitamin C and phenolics from the fruit	
Mechanism	US waves create microscopic channels inside fruit tissue structure that ease moisture removal Limiting production cost	US-temperature competition is a main limitation Risk of over-saturation	
Cost—Equipment	US provides a rapid processing technique, limiting the production cost Non-destructive technology	Pretreatment may increase processing and labor costs Requires specialized equipment with transducers	
Safety	US is an eco-friendly, green technology that acts as an excellent substitute for heat-based, conventional technologies detrimental to product quality	Long-time cavitation noise may affect the human health of workers	
Tissue Damage	US pretreatment promotes expansion of intercellular pores	Damages to tissues and cells can occur from US treatment	

**Table 3:** The advantages and disadvantages of CP pretreatment of fruits and vegetables before drying.

Category	Advantages	Disadvantages	References
Drying Time	CP pretreatment can reduce drying time by 12–61% through surface etching and microstructure modification	Optimal treatment times are product-specific and excessive treatment can increase drying time Shorter drying times may compromise product quality	[90,100,101,102,103,104,105,106]
Energy Efficiency	Total energy consumption reduction of 26.30–40% despite additional CP energy input	Results vary non-linearly with treatment parameters, requiring careful optimization	
Quality—Color	Improved color preservation through enzyme inactivation and reduced browning PPO and POD activity reduced by 58–85%, extending shelf life	May induce non-enzymatic reactions in some products (e.g., apples)	
Quality—Texture	Retain their natural firmness and crunchiness	Can cause excessive hardening or softening depending on treatment	
Nutrient Retention	Better retention of phenolics, flavonoids, vitamin C (17.87–168.73% increase)	ROS/RNS can degrade heat-sensitive compounds like vitamin C (2.8–7% loss)	
Antioxidant Activity	Enhanced antioxidant capacity due to preserved bioactive compounds	Potential reductions in the total phenolic content and antioxidant capacity	
Rehydration	Improved rehydration ratio by 7.3–16.4% due to structural modifications	Excessive treatment time can reverse benefits, causing structure collapse	
Mechanism	CP improves effective moisture diffusivity by 18.75–43.7% through creation of microcracks and pore formation	Limited penetration for large-sized products, effectiveness varies with size	
Cost—equipment	Reduction in time directly translates to substantial savings in thermal energy (e.g., natural gas, electricity for heaters), leading to a 25% to 46% in overall SEC	The equipment required for generating CP (e.g., DBD systems) can be expensive, potentially outweighing the immediate benefits for small-scale processors	
	Compatible with existing drying systems with minimal modifications	Plasma equipment expensive and currently at laboratory stage	
Safety	Uses only ambient air, no chemical residues, environmentally sustainable	Generation of toxic species (NO_2_, O_3_) requiring gas purification systems	
Tissue Damage	Creates micro-fissures and surface etching on the cuticle and outer epidermis. This disruption effectively opens up the tissue, drastically reducing mass transfer resistance	Over-treatment can cause excessive cell wall and membrane damage, potentially leading to leakage of turgor pressure and internal components	

**Table 4:** The advantages and disadvantages of OD pretreatment of fruits and vegetables before drying.

Category	Advantages	Disadvantages	References
Drying Time	Reduces drying time by 15–40% * Creates microchannels for faster moisture removal Synergistic effect with hot air drying	Limited effectiveness beyond 45 min treatment Diminishing returns with excessive exposure Some fruits show minimal time reduction (e.g., pineapple, papaya)	[64,110,122,123,124,125,126,127,128,129]
Energy Efficiency	Lower drying temperature requirements Reduced overall energy consumption (10–30% saving) ** Shorter processing times = less energy	High US equipment power consumption Extended pretreatment time adds to energy costs Energy efficiency varies by fruit type and equipment	
Quality—Color	Better color retention due to lower temperatures Reduced enzymatic browning Preserved natural pigments	Potential color changes from cell damage Oxidative reactions during prolonged treatment Inconsistent results across different fruits	
Quality—Texture	Softer texture after rehydration Reduced shrinkage and case hardening Improved structural integrity in some cases	Cell wall breakdown causing texture deterioration Over-treatment leads to mushy texture Tissue damage affects final product quality	
Nutrient Retention	Better preservation of heat-sensitive vitamins Reduced degradation of bioactive compounds Higher retention of phenolic compounds	Nutrient leaching into pretreatment liquid ROS * generation during long treatments Loss of minerals (K, Mg, P) during pretreatment	
Antioxidant Activity	Enhanced extraction of antioxidant compounds Increased phenolic content availability Higher DPPH ** radical scavenging activity	Oxidative degradation during prolonged exposure Variable effects depending on fruit type Optimal time window narrow (20–45 min)	
Rehydration	Improved rehydration capacity (10–25% better) Faster water absorption due to microchannels Better texture recovery after rehydration	Over-treatment reduces rehydration ability Tissue damage affects water holding capacity Cell wall collapse prevents proper rehydration	
Mechanism	Acoustic cavitation creates microchannels Mechanical effects enhance mass transfer Sponge effect improves moisture removal	Cavitation can damage cell structure Limited penetration depth in dense tissues Frequency-dependent effectiveness	
Cost—equipment	Batch systems relatively affordable Low maintenance requirements Scalable to industrial size	High initial investment for industrial systems Specialized equipment needed for different applications Limited suppliers and technical support	
Safety	No chemical additives required GRAS status for food applications Non-thermal processing method	Potential contamination from transducer materials Acoustic noise pollution Requires safety protocols for operators	
Tissue Damage	Controlled microchannel formation beneficial Enhanced mass transfer through tissue Improved accessibility for drying	Excessive cavitation destroys cell walls Loss of structural integrity Texture deterioration in final product	

* OD reduces subsequent drying time but requires its own processing time; ** syrup regeneration costs often offset OD energy savings.

**Table 5:** The advantages and disadvantages of HPP pretreatment of fruits and vegetables before drying.

Category	Advantages	Disadvantages	References
Drying Time	Significant reduction: Increases effective moisture diffusivity D_eff_ by creating micro-channels; reduces drying time by 15–40% depending on the matrix.	Requires specific optimization of pressure and holding time to avoid over-compaction of the tissue.	[127,130,148,149,150,151,152,153]
Energy Efficiency	Higher total efficiency: Shortened drying cycles lead to lower overall energy consumption despite the electrical input for the HPP unit Energy saving 5–15%	High specific energy input for the HPP unit itself during the initial compression phase	
Quality—Color	Superior retention: Preserves natural pigments (chlorophyll, carotenoids) and inhibits browning by inactivating PPO and POD enzymes	Possible “whitening” or opacity changes due to air-pocket compression or protein denaturation	
Quality—Texture	Structural control: Can produce a firmer texture in some vegetables (e.g., carrots) by activating PME	Pulse softening: High pressures often lead to a loss of turgor and softening of delicate tissues (e.g., strawberries)	
Nutrient Retention	High preservation: Covalent bonds (vitamins/minerals) remain intact; significantly better Vitamin C retention than thermal blanching	Some water-soluble nutrients may leach out if HPP is performed in a water medium without vacuum packaging	
Antioxidant Activity	Enhanced extractability: Disruption of cell walls increases the bioaccessibility of phenolic compounds and antioxidant capacity	Possible degradation of specific sensitive antioxidants during long-term storage if enzymes are only partially inactivated	
Rehydration	Improved ratio: Increased porosity and preserved cellular skeleton allow for faster and more complete water uptake	Excessive pressure can cause “structural collapse,” leading to poor rehydration and a “chewy” texture	
Mechanism	Isostatic permeabilization: Uniform pressure delivery ensures consistent treatment regardless of product shape/size	Complex interaction between pressure, temperature, and time (multi-parameter optimization needed)	
Cost—equipment	High throughput: Once installed, it allows for high-volume, continuous batch processing	Extreme capital cxpenditure: HPP units are among the most expensive food processing technologies	
Safety	Microbial inactivation: Effectively kills vegetative pathogens (*Listeria*, *Salmonella*) and spoilage yeasts/molds	Spore resistance: Does not inactivate bacterial spores (e.g., *Clostridium*) without the addition of mild heat.	
Tissue Damage	Intentional permeabilization: Controlled damage to cell membranes facilitates mass transfer (electroporation-like effect)	Irreversible damage: Can lead to “leakage” of intracellular fluids, potentially affecting the flavor profile of some fruits	

**Table 6:** The advantages and disadvantages of FT pretreatment of fruits and vegetables before drying.

Category	Advantages	Disadvantages	References
Drying Time	Creates large pores/voids that increase D_eff_; reduces drying time by 10–50% in fruits like blueberries and mangoes	Impact depends heavily on freezing rate; slow freezing creates larger, more effective crystals than fast freezing	[25,161,167,168,169,170,171,172]
Energy Efficiency	The energy saved during the shorter drying phase often outweighs the energy used for freezing Energy saving < 10%	The cumulative energy of freezing, thawing and drying must be calculated; not always efficient for small batches	
Quality—Color	FT preserves color better than hot-air blanching due to lower thermal exposure	The rupture of cells allows enzymes (PPO) and substrates to mix, leading to browning during the thawing phase if not controlled	
Quality—Texture	Post-drying, FT-treated products often exhibit a “puffy” and crisp texture due to internal voids	Fresh-like firmness is completely lost; the product becomes soft and “mushy” immediately after thawing	
Nutrient Retention	Avoids high-temperature degradation of heat-sensitive vitamins (e.g., Vitamin C)	Significant loss of water-soluble vitamins and minerals occurs in the “drip” liquid during the thawing process	
Antioxidant Activity	Cell wall rupture makes antioxidants like anthocyanins and phenolics easier to extract/digest	Increased surface area and cell damage can lead to rapid oxidation of antioxidants if drying doesn’t start immediately	
Rehydration	The highly porous “honeycomb” structure allows for very rapid water uptake compared to untreated samples	High rehydration can sometimes lead to a “slushy” or over-softened texture in the final rehydrated product	
Mechanism	Ice crystal growth exerts physical pressure that bypasses the need for chemicals or electricity	Damage is widespread and can sometimes destroy the structural integrity required for certain product shapes	
Cost—equipment	Uses standard industrial freezers; no need for specialized high-voltage (PEF) or high-pressure (HPP) units.	Requires significant freezer storage space and extra handling time for the thawing stage	
Safety	A purely physical process with no chemical additives or residues	The thawing phase (if done slowly at room temp) is a high-risk window for microbial proliferation in damaged tissues	
Tissue Damage	Essential for breaking down “skin” barriers (e.g., in grapes or blueberries) that otherwise resist drying	If freezing is too slow, the tissue can lose all structural identity, making it unsuitable for premium sliced products	

**Table 7:** The various pretreatment technologies for fresh-cut fruits and vegetables.

Pretreatment Technology	Primary Mechanism(s)	Impact on Quality & Bioactive Retention	Mitigation of Browning	Control of Cellular Leakage	Energy & Sustainability Benefits	Industrial Status & Scale-Up	Recommended Product Categories
PEF	Electroporation; creates nano- to micrometer pores in cell membranes	High retention of carotenoids (up to 80%+) and Vitamin C; preserves natural color	Inactivates enzymes via structural denaturation at high intensities (>10 kV cm^−1^). At lower intensities, it may trigger “metabolic defense” producing more antioxidants	Controlled electroporation allows for “gentle” leaching, which can actually remove surface sugars that contribute to Maillard browning during drying.	Very low energy input (1–10 kJ kg^−1^) vs. high savings (500–2000 kJ kg^−1^)	Pilot-scale; growing interest for drying applications	Berries & soft fruits: enhances permeability without heat. Thin-skinned tubers
US	Acoustic cavitation; creates microstreaming and cell wall breakdown	Enhances rehydration capacity and maintains open pore structures; improves antioxidant retention	Acoustic cavitation can detach PPO enzymes from the surface. When used with citric acid, US drives the inhibitor into the tissue.	The “sponge effect” keeps micro-channels open, preventing the surface from “sealing” with leaked cellular debris (case hardening).	Reduces drying time by 20–55%; non-thermal saves nutrients	Emerging industrial use, often in combination with other methods	Fibrous fruits/roots: apples, carrots, and potatoes where pore structure is critical
CP	Reactive species generation; surface etching/dewaxing and enzyme oxidation	Increases phenolic content (up to 45%); significantly reduces enzymatic browning (e.g., 52% HMF reduction)	Directly cold-denatures surface enzymes through reactive oxygen/nitrogen species (RONS) bombardment.	CP modifies surface tension (hydrophilicity), allowing leaked fluids to spread and evaporate faster rather than forming a sticky film.	Minimal to no water usage; effective at lower temperatures	Pilot scale; challenges in treatment uniformity and equipment design	Waxy/cuticular surfaces: blueberries, peppers, and grapes (dewaxing). Enzyme-sensitive cuts
OD	Osmotic pressure gradients; water removal through solute exchange	Modifies taste profile (sugar/salt uptake); reduces non-enzymatic browning	Physical oxygen barrier + solute infusion	Structural reinforcement via “solid gain” Dense and firm	Solution recycling possible; reduces initial moisture load for dryers	Widely used industrially, especially for tropical fruits	Tropical fruits: mangoes, pineapples, and papayas (infusion adds value)
HPP	High pressure (400+ MPa); protein unfolding and tissue density changes	Effective microbial/enzyme inactivation; changes tissue porosity and shrinkage	Highly effective at pressure-induced enzyme inactivation without heat, maintaining the “fresh” color of the cut edge.	Compacts the cellular matrix, reducing the “bleeding” of juice from the cut cells.	High capital investment; typically batch-processed	Commercially used for safety; limited application specifically for drying	Premium functional foods: bioactive-rich pulps and high-value fruit slices
FT	Ice crystal formation; mechanical stress and permanent structural damage	Creates highly porous structures; improves crispness and reduces shrinkage	Low-temp stabilization + phase bypass	Control of cooling rate (fast = low leakage) Porous and brittle	Enhances mass transfer via mechanical damage	Effective for specific textures (e.g., carrot slices)	Dense roots & waxy berries: carrots, blueberries. Contraindicated for leafy greens

## Data Availability

The original data presented in the study are openly available from the published literature and major electronic databases, including Web of Science, Scopus, and Google Scholar.

## Supplementary Materials

The following supporting information can be downloaded at: https://www.mdpi.com/article/10.3390/foods15030568/s1, Figure S1. Schematic diagrams illustrating the physical mechanisms of Pulsed-Electric Field (PFF); Figure S2. Schematic diagrams illustrating the physical mechanisms of Ultra-Sound (US); Figure S3. Schematic diagrams illustrating the physical mechanisms of Cold Plasma (CP); Figure S4. Schematic diagrams illustrating the physical mechanisms of Osmotic Dehydration (OD); Figure S5. Schematic diagrams illustrating the physical mechanisms of High-Pressure Processing (HPP); Figure S6. Schematic diagrams illustrating the physical mechanisms of Freeze-Thaw (FT).

## Abbreviations

The following abbreviations are used in this manuscript:

NTP	Non-Thermal Pretreatment
TP	Thermal Pretreatment
PEF	Pulsed electric field
US	Ultrasound-assisted treatments
CP	Cold plasma
OD	Osmotic dehydration
HPP, HHP	High-pressure processing
FT	Freeze-thaw
SEC	Specific Energy Consumption
PPO	Polyphenol oxidase
POD	Peroxydase
DBD	Dielectric barrier discharge
IMO	Isomalto-oligosaccharide
ROS	Reactive oxygen species
RNS	Reactive nitrogen species
DPPH	2,2-diphenyl-1-picrylhydrazyl
GRAS	Generally Recognized as Safe
PME	Pectin methylesterase
LNI	Liquid nitrogen immersion
CAPEX	Capital Expenditure
HMF	5-Hydroxymethylfurfural
TRL	Technology Readiness Levels
AD	Subsequent air-drying
D_eff_	Effective Moisture Diffusivity
DR	Drying (dehydration) rate
RR	Rehydration rate
US-CMC	Ultrasound-carboxymethyl cellulose
CAGR	Annual growth rate
HoReCa	Hotel/Restaurant/Café
RTE	Ready-to-eat
VPD	Vaccum-pulse drying
EPD	Explosion puff drying
ALI	AL- integration
EPD	EPD
