# Supplementary material for: Nonthermal Pretreatment Technologies to Improve Drying Efficiency and Quality in Fresh-Cut Fruits and Vegetables: A Comprehensive Review
Source: Foods. 2026 Feb 5;15(3):568. doi: 10.3390/foods15030568 (PMC12896815; doi:10.3390/foods15030568)
Supplement: Supplementary file 1 [file foods-15-00568-s001.zip › foods-4132629-supplementary.pdf]

Pretreatment Schematic Diagrams

Physical Mechanisms of Food Processing Technologies

1. PULSED ELECTRIC FIELD (PEF)

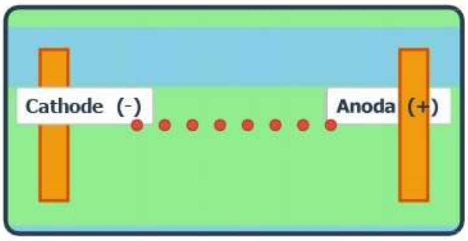

Physical Mechanism:

PEF applies short, high-voltage pulses (typically 10-80 kV/cm) across food materials placed between electrodes. The electric field induces transmembrane potential, causing reversible or irreversible electroporation of cell membranes. This creates pores that increase cell permeability, enhancing mass transfer, extraction efficiency, and microbial inactivation while preserving food quality attributes. The process is non-thermal and occurs within microseconds to milliseconds.

Figure S1. Schematic diagrams illustrating the physical mechanisms of Pulsed-Electric Field (PFF)

2. ULTRASOUND (US)

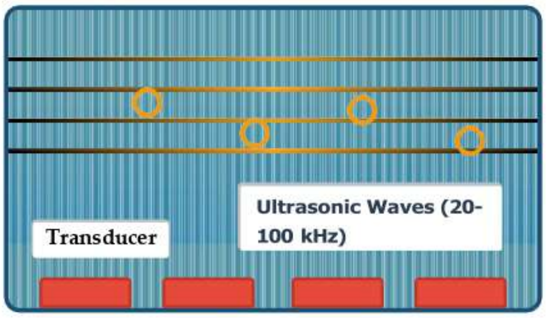

Physical Mechanism:

Ultrasound generates high-frequency mechanical waves that create alternating compression and rarefaction cycles in liquid media. During rarefaction, microbubbles form and grow through cavitation. These bubbles collapse violently during compression, producing localized high temperatures (5000 K), pressures (1000 atm), and intense shear forces. The phenomenon generates microstreaming, free radicals, and mechanical disruption of cell structures, enhancing mass transfer, extraction, and microbial inactivation.

Figure S2. Schematic diagrams illustrating the physical mechanisms of Ultra-Sound (US)

3. COLD PLASMA (CP)

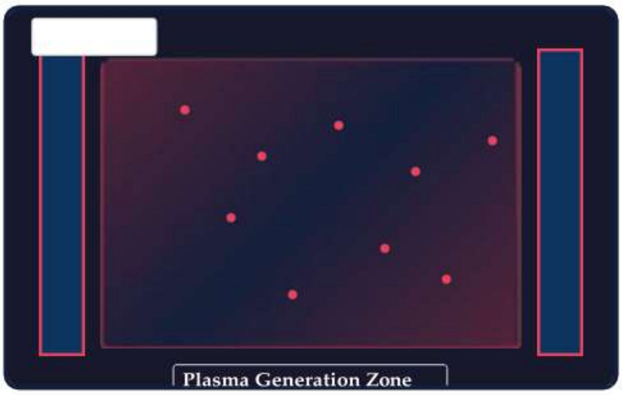

Physical Mechanism:

Cold plasma is generated by ionizing gas (air, oxygen, nitrogen) using electrical discharges at atmospheric or reduced pressure. The process creates a partially ionized gas containing reactive species including electrons, ions, radicals (OH•, O•, NO•), excited molecules, and UV photons. These reactive species interact with food surfaces, causing oxidative damage to microbial DNA, proteins, and lipids. The process operates at near-ambient temperatures (30 – 60 °C), making it suitable for heat-sensitive foods while providing surface sterilization and modifying food properties.

Figure S3. Schematic diagrams illustrating the physical mechanisms of Cold Plasma (CP)

4. OSMOTIC DEHYDRATION (OD)

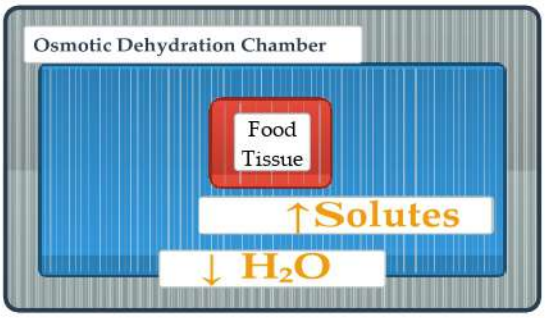

Physical Mechanism:

Osmotic dehydration involves immersing food materials in hypertonic solutions (sugars, salts) with higher osmotic pressure than the food's internal cellular fluids. Water migrates from the food tissue into the solution due to osmotic pressure gradient, while solutes from the solution penetrate into the food. This dual mass transfer results in water removal, solid gain, and concentration of natural components. The process reduces water activity, enhances product stability, modifies texture, and preserves color and flavor without thermal degradation.

Figure S4. Schematic diagrams illustrating the physical mechanisms of Osmotic Dehydration (OD)

5. HIGH-PRESSURE PROCESSING (HPP)

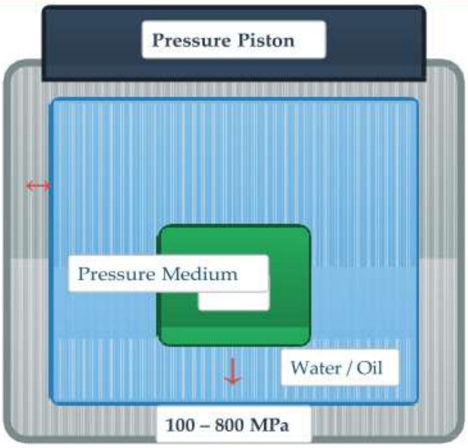

Physical Mechanism:

HPP subjects packaged food to hydrostatic pressures of 100 – 800 MPa using a pressure-transmitting medium (usually water). According to Le Chatelier's principle, pressure affects volume-dependent reactions. High pressure disrupts non-covalent bonds in proteins (denaturation), damages cell membranes, and inactivates microorganisms by affecting their cellular structures. Covalent bonds remain intact, preserving flavor, color, and nutritional quality. The process is isostatic (uniform pressure distribution) and occurs at ambient or low temperatures.

Figure S5. Schematic diagrams illustrating the physical mechanisms of High-Pressure Processing (HPP)

6. FREEZE-THAW (FT) PRETREATMENT

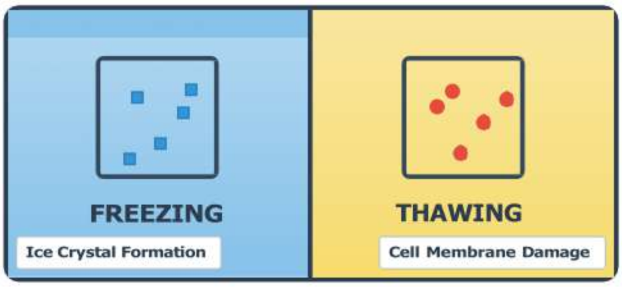

Physical Mechanism:

Freeze-thaw pretreatment cycles involve freezing food materials (typically -18 °C to -40 °C) followed by thawing at ambient or controlled temperatures. During freezing, water inside cells forms ice crystals that grow and disrupt cellular structures, membranes, and cell walls. Upon thawing, these crystals melt, leaving behind damaged tissues and increased porosity. The process creates micro- channels and fractures that enhance permeability, facilitating improved mass transfer, extraction efficiency, and drying rates while modifying texture and functional properties.

Figure S6. Schematic diagrams illustrating the physical mechanisms of Freeze-Thaw (FT)
